# Plants—Microorganisms-Based Bioremediation for Heavy Metal Cleanup: Recent Developments, Phytoremediation Techniques, Regulation Mechanisms, and Molecular Responses

**DOI:** 10.3390/ijms23095031

**Published:** 2022-05-01

**Authors:** Anas Raklami, Abdelilah Meddich, Khalid Oufdou, Marouane Baslam

**Affiliations:** 1 Laboratory of Microbial Biotechnologies, Agrosciences, and Environment, Labeled Research Unit-CNRST N°4, Faculty of Sciences Semlalia, Cadi Ayyad University, Marrakesh 40000, Morocco; anas.raklami@gmail.com (A.R.); oufdou@uca.ac.ma (K.O.); 2 Center of Agrobiotechnology and Bioengineering, Research Unit Labelled CNRST (Centre Agro-Biotech URL-CNRST-05), “Physiology of Abiotic Stresses” Team, Cadi Ayyad University, Marrakesh 40000, Morocco; a.meddich@uca.ma; 3 Laboratory of Agro-Food, Biotechnologies and Valorization of Plant Bioresources (AGROBIOVAL), Faculty of Science Semlalia, Cadi Ayyad University, Marrakesh 40000, Morocco; 4 Laboratory of Biochemistry, Faculty of Agriculture, Niigata University, Niigata 950-2181, Japan

**Keywords:** bioremediation, decontamination, mechanisms, phytoremediation techniques, plant-microbe association, contaminants, heavy metals, uptake and translocation

## Abstract

Rapid industrialization, mine tailings runoff, and agricultural activities are often detrimental to soil health and can distribute hazardous metal(loid)s into the soil environment, with harmful effects on human and ecosystem health. Plants and their associated microbes can be deployed to clean up and prevent environmental pollution. This green technology has emerged as one of the most attractive and acceptable practices for using natural processes to break down organic contaminants or accumulate and stabilize metal pollutants by acting as filters or traps. This review explores the interactions between plants, their associated microbiomes, and the environment, and discusses how they shape the assembly of plant-associated microbial communities and modulate metal(loid)s remediation. Here, we also overview microbe–heavy-metal(loid)s interactions and discuss microbial bioremediation and plants with advanced phytoremediation properties approaches that have been successfully used, as well as their associated biological processes. We conclude by providing insights into the underlying remediation strategies’ mechanisms, key challenges, and future directions for the remediation of metal(loid)s-polluted agricultural soils with environmentally friendly techniques.

## 1. Introduction

The accelerated population growth and the rapid urbanization, along with flourishing industrialization, have improved the living standards; however, as a consequence, an array of harmful pollutants (e.g., pesticides, hydrocarbon, heavy metals (HMs), microplastics) have been released and compromised the environmental quality (soil, air, and water) [1,2]. Due to their physicochemical properties, such as ubiquity, non-biodegradability, toxicity, accumulation, and persistence, HMs pollution has attracted much attention worldwide and has led to the recognition and understanding of the bond between HM contamination and public health [3]. Human socioeconomic and development activities, such as mining, smelting, fuel and energy use, long-term use of mineral fertilizers and pesticides, sewage sludge, and wastewater disposal, are the primary sources of HM contamination [3,4]. HM soil pollution has led to: (i) damage to soil quality and fertility, (ii) loss of microbial biodiversity, (iii) destruction of the vegetal cover, and (iv) a reduction of crop production and quality [5,6]. However, microorganisms and plants have developed morphological, physiological, metabolic, and molecular traits to cope with HM toxicity, and these traits could be used to remediate soil contaminated with HMs [7,8].

Several techniques have been deployed to remediate HMs in soils and restore soil characteristics as close as possible to pre-pollution conditions, including physical, chemical, and biological strategies [3]. The appropriate remediation strategies depend on the site characteristics, contamination degree, type of contaminants, and the final use of the contaminated soil. In general, the physicochemical techniques unduly focus on removing and stabilizing HMs in the soil [3]. Metal remediation through standard physicochemical techniques is simple, fast, efficient, and operative; however, these techniques are expensive, unsuitable for large contaminated areas, energy requirements, generate significant amounts of toxic sludge, and are not practical with a low metals content nor are they environmentally friendly [7,8]. In addition, these techniques may deteriorate the soil’s physicochemical characteristics and hamper its microbial biodiversity, which renders the soil unsuitable for agriculture and other uses [3,8]. To tackle these issues and efficiently remediate soils contaminated with HMs, scientists have established alternative biological-based strategies encompassing techniques that use microorganisms and techniques that exploit the plant properties [3,8,9]. Microbial remediation involves using microorganisms (e.g., bacteria, microalgae, yeast) to remove/immobilize, transform, or detoxify HMs from the environment [10,11,12,13]. While in phytoremediation, the use of plants and associated soil microbes involves the extraction, stabilization, and volatilization of HMs from the contaminated substrates [14,15,16]. The biological strategies exploit the mechanisms used by endogenous or exogenous microorganisms or plants to cope with HMs, such as extracellular/intracellular sequestration, production of metal chelators, precipitation, enzymatic detoxification, and volatilization, to remediate HM-contaminated environments [12,13,17,18,19]. These biological-based strategies are preferred over physicochemical ones because they are simple, easy to implement, could be adopted for large areas, reliable, cost-effective, less or non-destructive, and ecofriendly [3,8,9,20]. Nevertheless, there are still several bottlenecks in their wide application. Biological-based strategies depend on the type of microorganisms and plants used, their resistance, the level of contamination, and their soil physicochemical characteristics [3,8,9]. However, these limitations could be surpassed by designing new microbial and plant species that express specific genes of interest or combining microbial and/or phytoremediation with physicochemical strategies. Accordingly, this review addresses the accomplishment of plants’ uses and microorganisms’ application as tools for bioremediation of HM-contaminated environments. We also examine the recent developments and prospects for microbial remediation and phytoremediation of toxic pollutants.

## 2. Heavy Metals: An Alarming Threat to Soil and Environment

Environmental pollution is among the most critical environmental concerns and is considered to be one of the significant challenges in the modern era. Heavy metals (approximately 65 metals) are becoming the most threatening contaminants in the environment [21]. The term HMs refers to chemical elements with a density greater than 5 g/cm^3^ or specific gravity of at least five times greater than that of water and an atomic mass of over 20 u [21,22,23]. From a biological perspective, HMs could be classified into essential and toxic elements. Essential metals (in some cases metalloids) describe a series of plant and animal micronutrients, such as Zn, Fe, Ni, and Cu, but above certain thresholds, they become toxic [24]. In contrast, toxic elements are non-essential metals that are highly toxic even at low concentrations [20,23]. These metals (essential and non-essential) are omnipresent-trace elements in all the environmental compartments, but due to anthropogenic activities (i.e., urbanization, industrialization, mining, agriculture, and smelting), they accumulate in a particular site [25,26].

Due to their ubiquitous nature, not being biodegradable, toxicity, accumulation, and persistence, elevated HMs in soil and environment have been attracting much attention worldwide [27]. The adverse effects of HMs on soil physicochemical, biological, and biochemical properties are well-documented [5,27,28]. Moreover, the coexistence and persistence of HMs in the soil are also responsible for the entrance of toxic metals into the food chain, and thus lead to severe health hazards in living beings [25,29,30]. HMs drastically disorder soil horizons, soil structure, soil fertility, nutrient biogeochemical cycles, and soil microbial populations, destroying the existing soil vegetation and installing subsequent vegetal cover [6,31]. HMs indirectly affect soil enzymatic activities by altering the microbial community’s size, composition, and activity [32]. HMs exhibit toxic effects on the microbial community by affecting key microbial metabolic processes such as respiration, denitrification, and enzymatic activity [32,33,34]. HMs also cause a reduction in the number of specific microbial populations and a shift in the microbial community structure [32]. Furthermore, HMs inhibit microbial cell division, transcription, and protein denaturation and adversely affect cell membrane distribution [35]. The magnitude of the effects of metals on soil biological properties depends on several factors, including soil texture, clay contents, organic matter content, pH, inorganic anions and cations, and chemical forms and speciation of the metal [36].

HM pollution drastically affects soil quality and fertility and plant quality and yield. HMs severely hamper several biochemical and physiological processes in plants, such as seed germination, water balance, photosynthesis, electron transport system, stomatal conductance, as well as CO_2_ assimilation, antioxidant scavenging enzymes, solute accumulation, mineral nutrition, and stunted growth, and ultimately, these disorders could lead to plant death [31,37,38,39,40]. Moreover, high metal toxicity obstructs cytoplasmic enzymes in plant cells and causes damage to cell structures owing to oxidative stress, which consequently affects plant growth and metabolism [41,42]. The continued decline in plant growth reduces yield, leading to food insecurity.

HMs that have been extensively accumulated in soils could be infiltrated into other environmental compartments, such as groundwater, rivers, and crops, and therefore are menacing to human beings [43]. Researchers have demonstrated that HMs that surpass the permissible limits deteriorate water quality and make it unfit for drinking and irrigation purposes [44,45]. HMs enter the human body through direct ingestion or contact with a contaminated environment, the food chain, and drinking contaminated water [29,46]. Enduring levels of ingestion of these metals has a deleterious influence on humans, and the related dangerous impacts become noticeable after several years of exposure [29]. The toxicity degree of each metal is determined by the exposure duration and the absorbed dose.

The toxic effect of HMs and their permissible limits are listed below (Table 1).

**Table 1:** Maximum permissible limits of heavy metals and other pollutants in irrigation water, soils, and vegetables [47].

Chemical Element (Contaminants)	Maximum Permissible Level in Irrigation Water (µg/mL)	Maximum Permissible Level in the Soil (µg/g)	Maximum Permissible Level in Vegetables (µg/g)
As	0.001	20	-
Cd	0.0003	0.8	0.10
Co	0.05	50	50
Cr	0.55	100	-
Cu	0.017	36	-
Fe	0.02	50,000	425
Mn	0.04	2000	500
Ni	0.002	35	67
Pb	0.001	85	0.30
Se	0.02	10	-
Zn	0.20	50	100

## 3. Current Bioremediation Technologies: Status, Pitfalls, and Drawbacks

With the rapid urbanization and industrialization around the world, the cases of soil contaminated by HMs have led to the recognition and understanding of the bond between environmental contamination and public health [3,29,45]. Thus, the overall objective of any soil remediation strategy is to restore the contaminated sites to a condition as close as possible to pre-pollution conditions, protect human health, and preserve a sustainable environment for future generations [3,4]. Generally, remediation of heavy metal-polluted soils is subjected to an array of regulatory requirements and could also be based on human health assessment and ecological risks [30,48]. Due to their long-term consequences to the environment, it is a worse scenario to leave HM-polluted soil un-remediated. The appropriate soil remediation techniques depend on the site characteristics, contamination degree, type of contaminants, and the final use of the contaminated soil [49]. The current remediation techniques adopted to solve the problem of soil contamination are based, generally, on two principles. The first is the complete removal of contaminants, and the second is the transformation of contaminants into less harmful forms using engineering technologies [4,8,50]. In situ and ex situ are the two approaches practiced for decontaminating soils affected by HMs [3,8,51]. Numerous physical, chemical, and biological clean-up techniques have been suggested, practiced, and evaluated for HMs’ remediation in contaminated or polluted areas [3,8]. Typically, the physicochemical clean-up techniques are equitably widespread. Although these techniques are seen as suitable for removing the HMs from the environment, they are challenging to apply, very expensive, not specific, not efficient in a particular situation, and can significantly alter the soil quality [7,8]. In this context, scientists developed new methods for the remediation of contaminated soils. Biotechnological methods are an emerging and innovative technology that demonstrates increasing opportunities for removal and restoration of HM-contaminated soils, as they are reliable, cheap, and feasible alternatives [3,8,9]. Hence, this review focuses on biotechnological remediation techniques, especially phytoremediation and microbial remediation.

Bioremediation is an innovative and promising waste management technique that uses living organisms to remove and neutralize contaminants [3,9,52]. Even though traditionally, bioremediation refers to the bacterial use (benchmark organisms) in the bioremediation process, nowadays, the bioremediation strategies are based on processes and the potential of almost all life forms. In addition to the ‘traditional’ microorganisms’ remediation, strategies including phytoremediation, mycoremediation, and zooremediation are emerging [8,9]. These organisms have developed and adopted different depolluting mechanisms such as biosorption, bioaccumulation, biotransformation, and biomineralization for their survival under HM-polluted soils, which could be used for ex situ or in situ bioremediation [9,13,19,32]. Ultimately, these organisms make the pollutant less toxic, immobilize it, or extract it [13,53]. These bioremediation strategies are preferred over other methods due to their cost-effectiveness and because they are less or non-destructive to the environment [8]. These bioremediation strategies are typically classified into two main categories: bioremediation techniques that exploit the plant properties and bioremediation techniques that use microorganisms.

### 3.1. Phytoremediation

A very promising solar-powered, low-cost, cost-effective, efficient, and green alternative technology is plant-based phytoremediation, which has already been used for years. The concept of phytoremediation was first introduced in 1983 [54]. Phytoremediation, also called agro-remediation, botano-remediation, or green remediation, is a generic term that refers to the use of plants to extract, reduce, transform, or immobilize contaminants (organic and inorganic) contained in soils, sediments, and groundwater [8,20,55]. Several plants can accumulate large quantities of metals in their vegetative and reproductive parts [6]. Phytoremediation appears to be the best approach to treat soils contaminated with low to medium metal levels and can be applied in combination with other traditional soil restoration approaches for effective contaminant removal [20,23,51,56]. The use of plants helps the concentration and/or degradation of soil pollutants and helps prevent erosion through the root network, thus limiting dispersal in the environment. They also make it possible to create a physicochemical environment favorable to developing the rhizospheric microflora capable of detoxifying the pollutants present in the soils [20,56,57].

**Figure 1:**
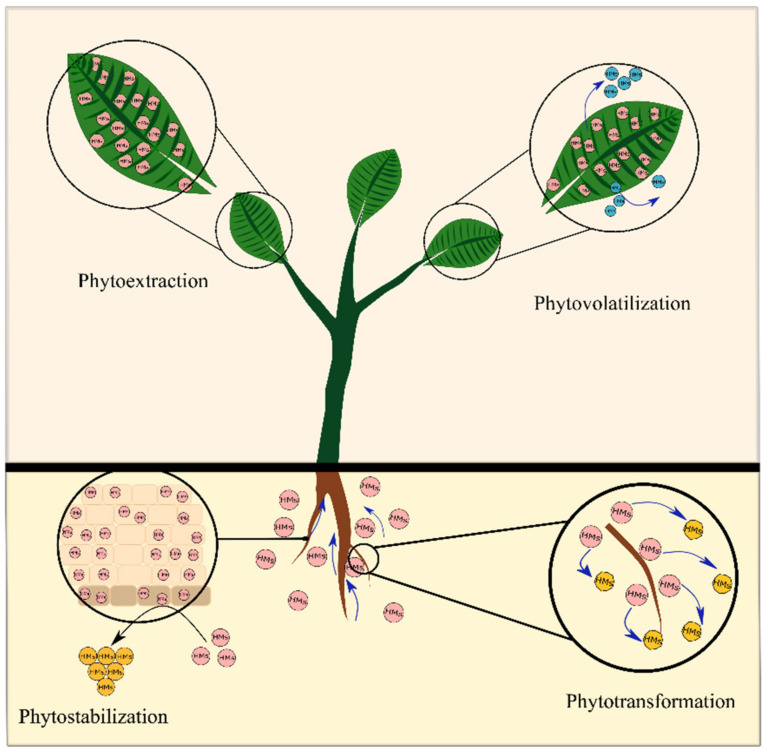
Main phytoremediation techniques for the remediation of contaminated environments. HM: Heavy metal.

Unlike physicochemical techniques, phytoremediation is environmentally friendly, inexpensive, and easier to implement. It can also be adopted in addition to physicochemical methods [31,58]. Phytoremediation technology of HM-contaminated soils is grouped into five sub-classes: phytoextraction, phytostabilization, phytovolatilization, phytotransformation, and phytofiltration [55,59] (Figure 1).

#### 3.1.1. Different Phytoremediation Technologies Involving Contaminants’ Removal

##### Phytoextraction

Phytoextraction, also known as phytoaccumulation or phytoabsorption, one of the in situ strategies, refers to using hyperaccumulating plants that could uptake metals from the soil by their roots and accumulate them in their aerial parts (stems and leaves) [35,55,56]. Phytoextraction is considered an efficient approach to removing HMs from contaminated soils without altering soil fertility (Figure 1). Plants used for phytoextraction are expected to have fast growth, high biomass, extensive root network, easy cultivation, repulsion to herbivores, and support and store HMs at high levels [20,35,56]. After harvest, metals can also be recovered and reused for economic benefits (phytomining) by thermal, chemical, or microbiological processes [20,60]. Practically, most hyper-accumulative plants are identified to belong to the Brassicaceae [23,57]. Several plants, such as *Brassica juncea*, *Thlaspi caerulescens*, *Pteris vittata*, *Haumaniastrum robertii*, *Aeolanthus biformifolius*, *Astragalus bisulcatus*, and *Arabis paniculate*, are identified as hyperaccumulator plants based on their maximum level of HM accumulation [61].

##### Phytostabilization

Phytostabilization (or phytoimmobilization) is an emerging alternative strategy involving establishing a plant cover on contaminated media [20,55,56]. Phytostabilization refers to the use of plants to immobilize contaminants in the soil through accumulation by the roots, adsorption on the roots, or precipitation in the root zone (due to binding by organic compounds), rendering HMs less soluble, less available, and less harmful to the environment [16,55,59] (Figure 1). Notably, this strategy is not a clean-up technique, but instead, it reduces the mobility of the contaminants and avoids the risk of further environmental contamination. Plants employed in phytostabilization require (i) tolerance to high levels of toxic contaminants, (ii) an extensive root system, and (iii) low translocation of toxic contaminants from the roots to the shoots [20,62]. The plants accumulate small amounts of HMs in the above-ground parts to avoid their transfer into the food chain. Metal-tolerant plant species can also be used to restore the vegetal cover to these sites and decrease the spread of toxic contaminants by wind, soil surface erosion, and leaching to the groundwater [38,62,63]. Therefore, this technique ensures better landscape integration of the contaminated sites. Recently, several leguminous plant species have been successfully used in phytostabilization owing to their rapid growth, high biomass, high metal tolerance, high metal accumulation in the root system, and their capacity to form a symbiosis with rhizobacteria [38,58,64]. Seminal works demonstrated the phytostabilization strategies in several plants, including *Medicago sativa*, *Vicia faba*, *Lens culinaris*, *Sulla coronaria*, *Trifolium repens*, *Acacia pycnantha*, *Mimosa caesalpiniaefolia*, *Erythrina speciose*, and *Schizolobium parahyba* [38,65,66,67,68]. *Medicago sativa* shows potential for stabilizing mine tailings contaminated with Cu, Zn, and Pb [31,58]. However, this strategy allows only the unavailability of HMs to organisms, but HMs remain in the soil. This technique aims to contain HMs in the soil when the site is heavily contaminated and the phytoextraction method seems inefficient [20].

##### Phytovolatilization

Phytovolatilization refers to the plant uptake of toxic contaminants from soil, their transformation into volatile products, and their subsequent discharge into the atmosphere [16,56,60] (Figure 1). It relies on the use of specific plants which uptake toxic contaminants such as mercury, selenium, and arsenic, transforming them into volatile elements with little or no toxicity and releasing them into the atmosphere by evapotranspiration via the stomata, leaves, or stems [16,20,59]. Generally, the released compounds are less toxic than the soil compounds taken up by the roots [16]. The main advantage of this strategy is removing the contaminants from the soil, without harvesting the plant, compared to other cases [69]. In contrast, this strategy allows the complete clean-up of the toxic compounds from soil, but they are released into the atmosphere, where they might be redeposited [59,70]. Sakakibara et al. [71] demonstrated that the vapor released from the frond of *P. vittata* included arsenic, arsenite, and arsenate compounds. Their results suggest that *P. vittata* efficiently volatilizes As by eliminating ca. 90% of the accumulated As.

##### Phytotransformation

Phytotransformation or phytodegradation is limited to eliminating organic contaminants since HMs are not biodegradable [60,69]. Phytotransformation breaks down organic pollutants by using plants enzymes, and it does not depend on rhizospheric microorganisms [60,72] (Figure 1). Recently, Oladoye et al. [56] revealed plants’ capacity to degrade various organic pollutants, including pesticides, hydrocarbons, and chlorinated solvents. Enzymes suitable for phytodegradation are: (i) dehalogenase (transformation of chlorinated compounds), (ii) peroxidase (conversion of phenolic compounds), (iii) nitrilase (transformation of cyanated aromatic compounds), (iv) nitroreductase (conversion of explosives, e.g., 2,4,6-Trinitrotoluene (TNT), and other compounds), and (v) phosphating (transformation of organophosphate pesticides) [16,69]. *Cyperus alternifolius* has been found to degrade and remove ethanolamines from wastewater [73], while *Armoracia rusticana* can degrade benzophenone [74].

##### Phytofiltration

Phytofiltration refers to the approach that exploits plant biomass to remove contaminants, mainly HMs, from polluted water, effluent, and aqueous waste streams with a low level of contaminants [69,75,76]. Phytofiltration permits the removal of organic and inorganic pollutants from water by using plant roots (rhizofiltration), seedlings (blastofiltration), or plant shoots (caulofiltration) [76]. The ideal plant for phytofiltration should: (i) produce a substantial amount of root or shoot biomass, (ii) be able to accumulate and tolerate significant amounts of metals, (iii) have easy handling, (iv) grow under submerged conditions, and (v) have a minimum of secondary wastes that further require disposal [75,76]. In rhizofiltration, fast-growing terrestrial and aquatic plants can be used for the extraction of Pb, Cd, Ni, Cu, Cr, V, and radionuclides (uranium (U), caesium (Cs), and strontium (Sr)) [75,76]. Based on the pollutants’ nature, the rhizofiltration process may occur with phytoextraction, phytostabilization, or phytovolatilization processes [69]. Phytofiltration offers an economical and ecologic solution for the purification of wastewater.

#### 3.1.2. Plant Mechanisms for Metal Detoxification

**Figure 2:**
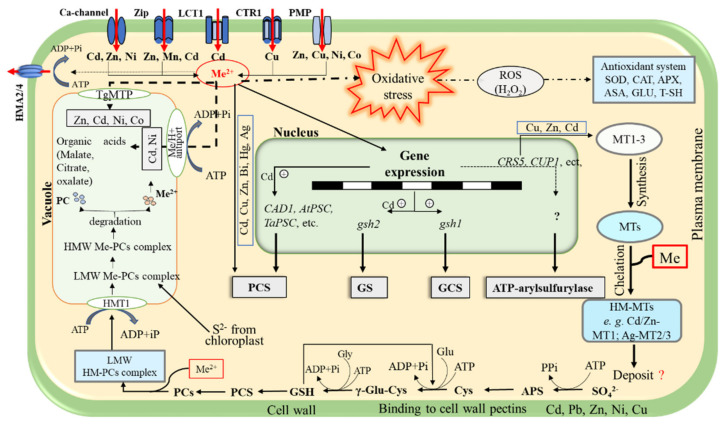
Schematic representation of various mechanisms involved in plant metal detoxification. ZIP, LCT1, and CTR1: metal transporters at the plasma membrane; HMA2/4: metal transporters; TgMTP and HMT: metal transporters at the tonoplast; Me^2+^: bivalent metals; ROS: reactive oxygen species; H_2_O_2_: hydrogen peroxide; SOD: superoxide dismutase; CAT: catalase; APX: ascorbate peroxidase; ASA: ascorbic acid; GLU: glutathione; T-SH: protein thiols; PCS: phytochelatin synthase; GS: glutathione synthetase; GCS: γ-glutamylcysteine synthetase; APS: adenosine 5’-phosphosulfate; Cys: cysteine; Glu: glutamate; GSH: glutathione; MT: metallothionein; LMW: low molecular weight; HMW: high molecular weight.

The plasma membrane, antioxidant system, intracellular chelation, and compartmentalization are the main mechanisms of plant tolerance and detoxification to HM stress [7,77,78] (Figure 2). The plasma membrane could play an essential role in the plant tolerance to HMs’ toxicity by limiting/reducing their uptake or modifying the ionic efflux [77,78]. HM homeostasis is mainly maintained by heavy metal ATPases (P_1_B–ATPase), natural resistance-associated macrophage protein (NRAMP), cation diffusion facilitator (CDF), and zinc-regulated transporters, iron-regulated transporter-like protein (ZIP) gene families’ transporters that are present on the plasma membrane [79] (Figure 2). Once in the cell, the HM accumulation at a particular stage of exposure leads to cell homeostasis disorder, affects DNA structure and function, causes damage to the chloroplast and pigments, and ultimately destroys the cell by producing reactive oxygen species (ROS) (e.g., O_2_^·−^, OH, RO_2_, and H_2_O_2_) [77]. The production of ROS can damage biomolecules such as lipids, proteins, and nucleic acids. Plants, in turn, use antioxidant enzymes, such as superoxide dismutases (SOD), catalases (CAT), peroxidases (POD), ascorbate peroxidase (APX), and glutathione reductase (GR), to scavenge ROS and reduce the oxidative damage caused during HMs’ oxidative stress [38,80]. One of the fundamental mechanisms of plant tolerance and detoxification of HMs is their chelation in the cytosol by specific ligands (Figure 2). Ligands such as organic acids, amino acids, phytochelatins, and metallothionein reduce HMs’ intracellular content and consequently their phytotoxicity [31,81]. Organic acids (fumaric, malic, oxalic, and citric acids) have been associated with HM tolerance in many plant species and have been recognized as critical cellular ligands for Zn, Cd, and Ni [81]. Indeed, these ligands are involved in the vacuole’s HM tolerance process, transport, and sequestration. Guo et al. [82] proved that the overproduction of organic acids (e.g., malate and succinate) is a response to excess cadmium uptake, and they act as a potential ligand of Cd, thereby reducing further damages. Amino acids (e.g., histidine, nicotianamine, and proline) have been described as HMs’ potential ligands in maize roots [77,79,83]. For instance, Kanwal et al. [83] showed an overproduction of proline by *M. sativa* during Zn and Cd exposure. As for microorganisms, the production of cytosolic polyphosphates and cysteine-rich proteins, including metallothionein, glutathione, and phytochelatins, which form insoluble metal precipitates, have been documented as a sequestration strategy in plants [79,84,85]. Talukder [85] has investigated the role of metallothionein and phytochelatins against Cu-induced oxidative stress in *Solanum melongena*. The results clearly showed that metallothionein and phytochelatins coordinate to detoxify HMs such as copper. Moreover, this study demonstrated that phytochelatins are more efficient HM chelators and detoxifiers than metallothionein and bind more HMs per cysteine residue. Cellular compartmentalization provides an effective strategy for HM tolerance and detoxification by removing HMs from the cell-sensitive sites (where cell division and respiration occur) to metabolically inactive cellular compartments, thus avoiding damage to the cell functions [77,79,86]. The vacuole represents the main inactive cellular compartment for HMs’ sequestration. Similarly, HMs can be sequestrated and compartmentalized into other locations, such as leaf petioles, sheaths, and trichomes [84,86].

#### 3.1.3. Phytoremediation: Drawbacks and Future Application

Phytoremediation is an effective biological technique for removing HMs from the soil, having the advantage of being an in situ approach. The installation of the vegetal cover conserves the topsoil and soil structure, and therefore protects the soil integrity [20,57,87]. In addition, it is recognized as an economical choice compared to conventional options since it requires less excavation, less equipment, and less labor. Phytoremediation is an eco-friendly approach owing to its benign role in ecosystem restoration and naturally preserving the environment. The public widely accepts this strategy [20,57,87]. Several other advantages are attributed to this remediation technique, including (i) improvement of soil fertility, (ii) amount of residue generated, (iii) it could be used for a wide range of organic and inorganic contaminants, and (iv) its effectiveness under soil and water contamination. The installation of the vegetal cover on the contaminated environment reduces or prevents the contamination of the surrounding area or groundwater and could improve and maintain the landscape aesthetics [31,62].

Phytoremediation is a viable remediation technology by using plants, and associated soil microbes, to reduce the content, or toxic effects, of contaminants in the environment, but it is not a ‘silver bullet’ and is still developing. The major downside to this technology is its slow process, which takes months to years to completely clean up the contaminated site [51,62]. Another limitation of this technology is that the depth of the root system dictates the extent of remediation possible, making this technology efficient only for the area covered by the depth reached by the roots [87]. Moreover, plant biomass plays an essential role in the extent of metals extracted [51]. Practically, low-biomass plants are not efficient for stabilizing and extracting toxic contaminants. Besides, climatic conditions, soil physicochemical characteristics, and biotic stresses may restrict plant growth and thus the bioremediation process. Absorbed contaminants can also present a potential risk to animal and human health since they can enter the food network (thus, the biomass needs proper handling and disposal) [3,60,62,87]. Therefore, the phytoremediation areas must be protected to prevent animal access. The plant age also determines the success of the overall process. As young plants tend to take up more metals than older ones, it is crucial to maintain a healthy plant population in the target site throughout the process [51,88]. The presence of high levels of HMs in the soil can induce phytotoxicity, so the level of contaminants plays an essential role in the applicability of this technology [31]. In the phytoremediation process, it is preferred to use native plants since introduced plants might affect biodiversity. Additionally, phytoremediation depends on many factors, such as the bioavailability of metals in the soil, soil properties, metal speciation, and the plant species involved [31,51,87].

### 3.2. Plant-Associated Microbe’s Affair for Environmental Clean-Up

The plant–microorganisms’ interaction is another approach for the remediation of HM-contaminated soils. At the ground level, the rhizosphere and microorganisms from particular microbial communities establish a mutual relationship, thus helping plants cope with contaminants, including HMs, to remedy the environment [55,84,86]. Several microorganisms, including rhizobacteria, mycorrhizae, and yeast, have been proposed as target inoculants to improve plant growth, fitness, tolerance, performance, and the phytoremediation process [31,38,89] (Table 2).

**Figure 3:**
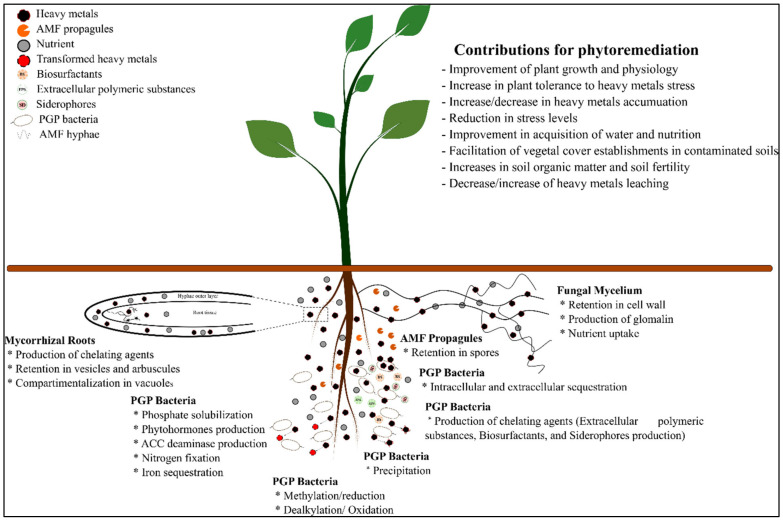
Microbial-assisted phytoremediation for heavy metal decontamination of polluted soils.

Among microorganisms, plant growth-promoting rhizobacteria (PGPR) have considerable potential to improve phytoremediation efficiency. PGPR can promote plant growth and fitness, improve plant nutrient uptake, protect plants against various abiotic and biotic stresses, and improve soil fertility and structure [84,86,90,91]. This is achieved through various direct and indirect mechanisms, including atmospheric N fixation, P and K solubilization, phytohormone production, and 1-aminocyclopropane-1-carboxylate (ACC) deaminase (Figure 3) [92,93,94]. Phytohormones such as auxin and cytokinin can stimulate cell division, cell enlargement, apical dominance, lateral root initiation, root hair development, and plant growth [95]. PGPR can produce ACC deaminase, which cleaves ACC (ethylene precursor in plants). The production of the ACC deaminase decreases the level of ethylene in the plant, its inhibitory effect (especially root elongation and plant growth in general), and thus improves plant growth under HM-stressed conditions. PGPR with P solubilizing activity could withstand as a strategy to mitigate HM stress (Figure 3). Through these mechanisms, plants’ health, fitness, and robustness are improved, facilitating the adaptation of plants under HMs’ stressful conditions [86,92,95]. PGPR can contribute to the phytoremediation process through metals’ solubilization, siderophores, organic and biosurfactants production, reduction/oxidation, methylation, precipitation, and biosorption (see Section 3.4.2: Mechanism of heavy metal remediation by microorganisms) that affect the bioavailability of metals in soils and sediments [7,96]. Inoculation of *M. sativa* with a bacterial consortium composed of four bacterial strains: *Proteus* sp. DSP1, *Pseudomonas* sp. DSP17, and two *Ensifer meliloti* RhOL6 and RhOL8 strains, improved seed germination, plant early growth, physiology, and attenuated HM stress by reducing the antioxidant enzymes and HMs’ accumulation content, ultimately improving the phytostabilization process efficiency [38,39].

Mycorrhizae can facilitate the phytoremediation process by making metals more bio-available or reducing their uptake [63,97]. However, improving phytoremediation is highly variable depending on the fungal species and the ecotype [97,98]. Mycorrhizae contribute to phytoremediation by retaining HMs on fungal mycelium (as a physical barrier) and their immobilization in the soil by gloaming, reducing their bioavailability, translocation, and bioaccumulation in the plant tissues (Figure 3) [97,98]. The immobilization of metals in the fungal hyphae (by chelation and sequestration) is probably the primary protective mechanism conferred on plants by mycorrhizae [63,98]. Liu et al. [99] demonstrated that inoculation of maize culture with *Glomus intraradices* improves plant growth and reduces the amount of Cd transferred to the aerial part. In *Nicotiana tabacum* roots, Janoušková et al. [100] demonstrated that AMF extraradical mycelium could accumulate 10 to 20 times more Cd per biomass unit than non-mycorrhizal plants. Some studies have shown that fungal spores, arbuscules, and vesicles could be involved in the HMs’ storage, thereby constituting additional protective mechanisms against metal toxicity [101]. However, mycorrhizae can also contribute to phytoextraction by increasing metal bioavailability, transfer from soil to roots, and translocation from roots to shoots [98,101]. Extraradical mycelium provides an uptake pathway for the different metals through its plasma membrane [102]. Handa et al. [103] have demonstrated that 3641 genes were differentially expressed, of which ca. 80% were upregulated, during arbuscular mycorrhizal development in *Lotus japonicus*. The upregulated genes included secreted proteins, transporters, proteins involved in lipid and amino acid metabolism, ribosomes, and histones. Production of chelating agents and metal transformation, passive or active transport from fungal cells to plant cells, are the major mechanisms involved in mycorrhizae-assisted phytoextraction [101].

**Table 2:** Microorganism-assisted phytoremediation of heavy metal (HM)-contaminated media.

Microbial Species	PGP Features	Plant	HMs	Main Results	Reference
*Proteus* sp., *Pseudomonas* sp., *E. meliloti, Glomus* sp., *Sclerocystis* sp., *Acaulospora* sp.	IAA, Biofilm, P solubilization, K solubilization	*M. sativa*	Cu, Zn, Pb	- Inoculation improved plant growth and proline content - Microbial inoculation decreased HM uptake.	[31]
*Streptomyces* sp.	IAA, ACCD, Zeatin, GA, P solubilization	*Zea mays*	As, Cr	- Inoculation increased germination traits, photosynthetic pigments, shoot and root tolerance indexes, FW and DM, leaf length, root and cob	[37]
*Proteus* sp., *Pseudomonas* sp., *E. meliloti*	IAA, Biofilm, P solubilization, K solubilization	*M. sativa*	Cu, Zn, Pb	- Inoculation alleviated metal stress, improved growth, lowered antioxidant enzymes’ levels, and increased physiological parameters. - Inoculation reduced the above-ground HMs.	[38]
*Aspergillus niger, Penicillium chrysosporium*	ACCD, IAA, Gibberellins P solubilization, siderophores	*V. faba*	Cd, Pb	- Inoculated plants modulated metal-induced oxidative stress by inhibiting metal transport and decreasing electrolyte leakage and lipid peroxidation. - Inoculation actively absorbed HMs and decreased their content in soil and plants.	[41]
*Glomus mosseae, Sinorhizobium meliloti*	n/s	*M. sativa*	Cd	- Single and combined inoculation improved alfalfa resistance to Cd stress. - Inoculation improved Cd tolerance via antioxidant enzyme activity increases	[42]
*Streptomyces pactum*	n/s	*Sorghum bicolor*	Zn, Pb, Cd, Cu	- Shoot and root DM and Chl content were improved after inoculation - Inoculation improved β-glucosidase, alkaline phosphatase, and urease activity in the soil and decreased antioxidant activity (POD, PAL, PPO) in plants. - Uptake of Zn (45%) and Cd (22%) was reduced, while that of Pb (17%) and Cu (47%) was increased.	[80]
*Bacillus* sp.	IAA, hydrolytic and ligninolytic enzymes, Siderophores	*Phragmites communis*	Fe, Cu, Zn, Cd, Mn, Ni, Pb, As	- In situ phytoremediation assisted by bacterial inoculation reduced HMs’ contents - *P. communis* showed higher potential for enriching Fe, Cu, Zn, and Cd in its rhizomes, roots, and shoots vs. leaves.	[96]
*Acinetobacter lwoffii*	IAA, ACCD, EPS, siderophore, P solubilization	*Vigna radiata*	As	- Inoculation increased growth, plant number per pot, and Chl and Caros content. - Inoculation decreased ROS production. - As uptake in plant tissues was reduced by inoculation.	[104]
*Aliinostoc* sp.	phosphatase production, N fixation	*Oryza sativa*	Cd	- *Aliinostoc* sp. promoted rice growth and yield through N input and P activation - *Aliinostoc* sp. immobilized Cd and decreased its uptake and grain translocation - *Aliinostoc* sp. binds with Cd through hydroxyl, carboxyl, and amino groups.	[105]
*Arthrobacter* sp., *Bacillus altitudinis, Bacillus megatherium, Sphingomonas* sp.	ACCD, IAA, Siderophore, P solubilization	*Brassica napus*	Cd	- The four PGPBs enhanced shoot biomass production, soil and plant analyzer development value, and Cd uptake and translocation to the leaves.	[106]
*Arthrobacter* sp., *Microbacterium oxydans*	IAA, ACCD, siderophores	*Noccaea caerulescens* and *Arabidopsis*	Ni, Cu, Co, Mn, Fe	- Single or dual inoculation was efficient in promoting *N. caerulescens* plant growth and biomass, soil Ni removal and phytoextraction, and Fe, Co, and Cu. - Inoculation stimulated root length, shoot biomass, and Ni uptake in Arabidopsis.	[107]
*Bacillus cereus, Pseudomonas moraviensis*	n/s	*Triticum aestivum*	Cd, Co, Cr, Cu, Mn, Ni, Pb	- Inoculation improved seed germination, FW, plant height, chlorophyll, flag leaf area, and tiller number per plant. - Inoculation decreased Cd, Co, Cr, Cu, Mn, Ni, and Pb accumulation in wheat	[108]
*B. cereus*	IAA, siderophores	*Zea mays*	Cd, Cu, Ni, Pb, Zn	- Inoculation enhanced the biomass, pigments, phenols, protein, and antioxidants. - Inoculation increased the translocation of HMs, except for Ni.	[109]
*Bacillus licheniformis, Micrococcus luteus, Pseudomonas fluorescens*	P solubilization, N fixation, siderophores	*Vitis vinifera*	As	- *M. luteus* increased plant biomass, protein content, and POX activity. - *B. licheniformis* increased plant biomass and APX. - *P. fluorescens* did not affect As but augmented POX activity.	[110]
*Bacillus megaterium*	IAA, arginine decarboxylase, siderophores	*Brassica campestris* and *Brassica rapa*	Cd	- Inoculation increased biomass, soluble proteins, and vitamin C content. - Inoculation decreased the edible tissue Cd and Pb content - Strain increased the OM content and invertase activity of the rhizospheric soils.	[111]
*Bacillus safensis* *Kocuria rosea*	n/s	*Helianthus annuus*	Cd, Fe, Zn	- Single or combined inoculation enhanced plant growth. - Inoculation increased Fe, Zn, and Cd uptake by plants. - Bacterial inoculation increased Cd uptake in the shoot by 30% - Bacterial inoculation increased the total biomass by 25%.	[112]
*Bacillus* sp., *Klebsiella* sp., *Leifsonia* sp., *Enterobacter* sp.	P solubilization, IAA and EPS production	*Z. mays*	Cd	- Bacterial strains increased plant growth and biomass in Cd-contaminated soil. - Cd uptake increased in plant tissues upon bacterial inoculation.	[113]
*Bacillus* sp., *Pseudomonas* sp., *G. mosseae*	IAA, HCN, siderophores, P solubilization	*Eucalyptus camaldulensis*	Cd	- Inoculation improved plant growth traits (shoot DM, height, and leaf area). - Bacterial inoculation increased Cd uptake by 90%. - Mycorrhizal inoculation increased Cd uptake by 24%.	[114]
*B. safensis*, *P. fluorescens*	ACCD, IAA Siderophore	*H. annuus*	Zn, Pb	- Inoculation reduced Zn and Pb uptake by plant tissues.	[115]
*Brevibacterium casei*	NH_3_, HCN, IAA, ACCD	*Sinapis alba*	Cd, Zn, Cu	- *B. casei* promoted plant growth and enhanced Cd, Zn, and Cu phytoextraction	[116]
*Chaetomium cupreum*	Siderophore	*Miscanthus sinensis*	Al, Cu, Fe, Pb, Zn	- Inoculation reduced HM uptake and promoted seedling growth and Al tolerance via inducing chlorogenic-acid and oosporein production.	[117]
*Chlorella vulgaris, Pseudomonas putida*	n/s	*O. sativa*	As	- Microbial consortium of *P. putida* and *C. vulgaris* improved growth and reduced As-induced oxidative stress in rice - Consortium reduced As accumulation and improved the mineral nutrient in rice.	[118]
*Debaryomyces hansenii*	IAA, P and Zn solubilization, Siderophores	*O. sativa*	As	- Yeast inoculation improved plant growth, total Chl, sugar, and proline. - Inoculation improved plant detoxification through ROS scavenging, - Rice–*D. hansenii* interaction reduced As in grain (40%).	[119]
*Funneliformis mosseae*	n/s	*Glycine max*	Cu, Pb, Zn	- AMF inoculum promoted soybean growth and seed yield. - Inoculation increased HMs’ retention in roots, and reduced shoot translocation of Cu (22%), Pb (58%), and Zn (67%).	[120]
*F. mosseae, Diversispora spurcum*	n/s	*Cynodon dactylon*	Pb, Zn, Cd	- *D. spurcum* inoculation increased Bermuda grass growth. - AMF inoculation increased soil pH and P, S, and HMs’ uptake by Bermuda grass, and decreased available Pb and Zn in soils and Pb in shoots.	[121]
*Glomus aggregatum, G. intraradices, Glomus elunicatum, Glomus versiforme*	n/s	*M. sativa*	Cd	- AMF inoculation promoted plant growth and contents of N and P in plant shoots - AMF reduced Cd uptake in plant tissues.	[122]
*G. versiforme*	n/s	*Solanum nigrum*	Cd	- Colonization enhanced soil acid phosphatase activity, P uptake, and growth. - *G. versiforme* increased Cd phytoavailability. - Inoculation improved the total Cd uptake in all plant tissues.	[123]
*Klebsiella oxytoca*	P solubilization	*H. annuus*	Co, Pb, Zn	- Inoculation enhanced plant growth under HM-contaminated soils. - Improvement of Co, Pb, and Zn phytoextraction by inoculation. - Inoculation improved Co (51%), Pb (20%), and Zn (76%) bioavailability.	[124]
*Klebsiella* sp.	IAA, EPS, NH_3_ P solubilization,	*V. radiata*	Cd, Cu, Pb	- Inoculation promoted plant growth under HM stress.	[125]
*Kocuria flava*, *Bacillus vietnamensis*	IAA, EPS siderophores	*O. sativa*	As	- Inoculation promoted plant growth (shoot and root length and weight). - As uptake by shoot and root was decreased.	[126]
*Oscillatoria* sp.	n/s	*Portulaca oleracea*	Cr, Fe, Al, Zn	- Cyanobacteria increased plant growth, Chl a, and N content. - Decreased the HMs’ extractable fraction and their accumulation in plant tissues.	[127]
*Oscillatoria* sp., *Leptolyngbya* sp.	n/s	*Lactuca sativa* and *Raphanus sativus*	Fe, As, Pb, Cr, Ni	- Inoculation into polluted soil increased root and hypocotyl lengths and vigor index due to high nutrient content and less HMs’ bioavailability.	[128]
*Paecilomyces formosus, Penicillium funiculosum*	IAA, gibberellins, P solubilization	*G. max*	Al, Ni, and Cd	- Inoculation promoted plant growth attributes, photosynthetic activity, macronutrient uptake, glutathione, CAT, and SOD activities, and decreased MDA. - Inoculation reduced metal accumulation and translocation in plants by downregulating HMs’ ATPase gene expression.	[129]
*Pantoea agglomerans, Bacillus aryabhattai*	ACCD, N fixation, P solubilization, siderophores	*Spartina densiflora*	As, Cu, Pb, Zn, Cd	- Inoculation decreased antioxidant enzymes vs. non-exposed plants. - Inoculation induced the expression of phenylalanine ammonium lyase.	[130]
*Pantoea stewartii*, *Microbacterium arborescens*, *Enterobacter* sp.	IAA, ACCD, siderophores	*Leptochloa fusca*	Cr, Cu, Fe, Ni, Pb, Ba, Cd, Co	- Bacteria enhanced *L. fusca* growth and helped in inorganic pollutants’ removal from the tannery effluent.	[131]
*Penicillium janthinellum*	IAA adsorption	*C. dactylon*	Cd	- Inoculation facilitated plant growth in the presence of Cd. - Inoculation increased Cd uptake in the stem and root of Bermuda grass.	[132]
*Piriformospora indica*	n/s	*Artemisia annua*	As	- Fungal colonization accumulated and immobilized As in the roots vs. aerial parts.	[133]
*P. indica*	n/s	*Cenchrus purpureus*	Cd	- Colonized roots promoted plant growth. - Inoculation increased Cd accumulation in plants.	[134]
*Planomicrobium chinense*, *B. cereus*, *P. fluorescens*	P solubilization	*Z. mays*	Ni, Cd, Pb, Co, Cu, Fe, Zn	- PGPR increased the maize plant’s root and shoot weight, root length, shoot height, leaf area, proline, chlorophyll, and carotenoid content. - PGPR induced Ni, Pb, Co, Fe, Cu, and Zn accumulation in maize shoot.	[135]
*P. aeruginosa*	IAA, HCN, NH_3_, ACCD siderophore, P solubilization	*T. aestivum*	Cu, Cr, Cd	- Inoculated wheat had better growth and yields under Cu, Cd, and Cr stresses. - Bioinoculant enhanced spikes, grain, and straw by 25%, 17%, and 12%, respectively. - Inoculation declined antioxidants and HMs’ uptake.	[136]
*P. aeruginosa, Actinomyces* sp., *Azotobacter* sp., *Azospirillum brasilense., Bacillus subtilis*	n/s	*Eichhornia crassipes*	As	- *Pseudomonas* and *Azotobacter* inoculation to *E. crassipes* enhanced As removal. - Co-inoculation with *Pseudomonas, Azotobacter, Azospirillum, Actinomyces*, and *Bacillus* consortium resulted in higher As phytoaccumulation efficiency.	[137]
*P. fluorescens*	IAA, ACCD, siderophore	*Sedum alfredii*	Cd	- Inoculation increased plant biomass, Cd, chlorophyll, and enzymes’ activity. - Inoculation improved the relative expression of *ZRT/IRT-like protein* gene family, *natural resistance-associated macrophage protein*, and HMs’ *ATPase*. - *P. fluorescens* stimulated *SaHMAs* (*SaHMA2*, 3, and 4) expression, which enhanced Cd root-to-shoot translocation - *P. fluorescens* upregulated *SaZIP* and *SaIRT1* to increase Cd uptake.	[138]
*P. fluorescens*	n/s	*Pisum sativum*	Pb	- Inoculation with Pb-tolerant PGPR strain immobilized Pb in soil and alleviated its harmful impacts on plant growth - Inoculation reduced Pb concentration in the roots and shoots.	[139]
*Pseudomonas libanensis*, *Claroideoglomus claroideum*	ACCD, IAA, P solubilization Siderophores	*H. annuus*	Ni	- Single inoculation or combined enhanced plant growth, physiological status (e.g., electrolyte leakage, chlorophyll, proline, and MDA), and Ni accumulation.	[140]
*Pseudomonas* sp.	IAA, EPS, HCN P solubilization, N fixation, siderophores	*M. sativa*	Cr	- Inoculation improved shoot (98%) and root (95%) DM. - Inoculation increased Chl content and decreased stress markers. - Inoculation decreased Cr content in the root.	[141]
*Pseudomonas* sp., *Serratia* sp.	Organic acids, ACCD, IAA, Acetoin, P solubilization, N fixation	*Helianthus tuberosus*	Cd and Zn	- Inoculation significantly enhanced the growth of *H. tuberosus*. - Inoculation decreased the content of Zn and Cd in the shoot and the root.	[142]
*Pseudomonas* sp., *Glomus* sp.	n/s	*Centaurea cyanus*	Pb	- Microbial inoculation increased shoot DM and Pb accumulation. - Bacterial inoculation enhanced shoot Pb concentration. - AMF increased plant biomass and plant Pb accumulation.	[143]
*Pseudomonas* sp., *Azotobacter* sp., *Paenibacillus* sp., *Streptomyces* sp. *Glomus* sp.	P solubilization Siderophores, IAA production	*Pennisetum glaucum* *S. bicolor*	Fe	- Bacterial and/or AMF inoculation enhanced plant growth and increased the extent of Fe absorption and phytoremediation efficiency.	[144]
*Rhizoglomus intraradices, Glomus etunicatum*	n/s	*T. aestivum*	As	- Mycorrhizal plants displayed better growth and less oxidative stress. - AMF increased As accumulation and reduced As translocation to grain. - Colonization of AMF resulted in higher antioxidant enzymes’ activities.	[145]
*Rhizophagus fasciculatus*, *Rhizophagus intraradices*, *F. mosseae G. aggregatum*	n/s	*Z. mays*	Cd, Cr, Ni, Pb, Fe, Zn, Cr, Mn	- AMF significantly influenced plant growth and phytoremediation potential. - AMF improved proline, chlorophyll content, and P content of shoot and root. - AMF inoculation improved the soil enzymes’ activity (dehydrogenase, β-Glucosidase, acid, and alkaline phosphatase).	[146]
*R. intraradices, G. versiforme*	n/s	*Z. mays*	Cd	- Inoculation enhanced biomass production and reduced Cd in shoots and roots. - AMF increased GSH and PCs contents in shoots and roots.	[147]
*R. irregularis*	n/s	*M. sativa*	Zn, Cd	- Inoculation improved plant growth, pigments, and g_s_ - AMF reduced Zn and Cd uptake in plant tissues.	[63]
*Rhodobacter sphaeroides*	IAA production	*T. aestivum*	Cd, Zn	- Inoculation reduced Cd (31%) and Zn (100%) exchangeable phases in soil. - Cd levels were reduced in wheat leaf (62%) and root (47%).	[148]
*Serratia* sp.	IAA production, P solubilization, ACCD	*H. annuus*	Cu, Zn, Ni, Pb, As	- Inoculation promoted plant height (40%) and root length (100%). - Inoculation enhanced Cu, Ni, Zn, Pb, and As rhizoaccumulation.	[149]
*Simplicillium chinense*	n/s	*P. communis*	Pb and Cd	- Inoculation enhanced the phytoextraction of Cd and Pb by *P. communis*.	[150]
*S. meliloti, P. fluorescence, P. indica*	IAA, HCN, ACCD P solubilization siderophores	*M. sativa*	Cd	- Inoculated alfalfa showed higher biomass and nutrient uptake. - Inoculation increased Cd uptake by alfalfa roots.	[151]
*Spirulina platensis*	n/s	*Z. mays*	Cd	- Microbial priming improved plant growth, photosynthetic electron flows, and non-photochemical quenching in Cd-exposed maize plants. - Cd translocation from root to shoot was significantly restricted.	[152]
*S. pactum*	n/s	*Lolium perenne*	Pb	- Inoculated plants had higher biomass, height, and root and tiller number. - Higher CAT, SOD, and POX activities in inoculated plants under Pb stress. - Inoculation increased Pb uptake and its phytoremediation.	[153]
*S. pactum, Bacillus* sp.	n/s	*B. juncea*	Cd, Pb, Cu, Zn	- Applying *S. pactum* promoted plant growth and metals’ uptake, and *Bacillus* sp. improved enzyme activity and metals’ availability. - Co-inoculation improved microbial community, enzymes’ activity, and growth. - Co-inoculation altered Cd, Cu, Pb, and Zn fractions, bioavailability, and phytoextraction.	[154]
*Talaromyces pinophilus*	Gibberellic acid	*T. aestivum*	Cd, Ni, Cu, Zn	- Inoculation with *T. pinophilus* boosted plant growth parameters, photosynthetic pigments, osmolytes, enzymatic antioxidants, and minerals (K, Ca, and Mg). - Inoculation reduced Cd, Ni, Cu, and Zn in the growth media, shoot, and root.	[155]
*Trametes hirsuta*	n/s	*T. aestivum*	Pb	- Fungal inoculation increased plant growth (+24%) and total chlorophyll (+18%). - Inoculation increased Pb uptake by plant tissues.	[156]
*Trichoderma asperellum*	n/s	*Suaeda salsa*	Pb	- *T. asperellum* promoted plant growth and alleviated oxidative plant damage. - Inoculation decreased Pb accumulation by plant tissues.	[157]
*Variovorax paradoxus, Rhizobium leguminosarum* *Glomus* sp.	n/s	*P. sativum* and *B. juncea*	Cd, Zn, Fe, Mn	- Inoculation increased plant biomass. - Inoculation decreased shoot Cd and increased seed Cd concentration of *P.sativum*, but had little effect on Cd concentration of *B. juncea*. - Inoculation increased Ca, Fe, K, Mg, Mn, N, P, S, and Zn accumulation under Cd-treated pea plants.	[158]

n/s: not specified. ACCD: 1-Aminocyclopropane-1-carboxylic acid deaminase, APX: ascorbate peroxidase, caros: carotenoids, CAT: catalase, Chl: chlorophylls, DM: dry matter, EPS: exopolysaccharides, FW: fresh weight, HCN: hydrogen cyanide, GA: Gibberellic acid, GPX: Glutathione peroxidase, GR: Glutathione reductase, g_s_: stomatal conductance, IAA: Indole-3-acetic acid, n/s: not specified, GSH: glutathione, PCs: phytochelatins, MDA: Malondialdehyde, PAL: Phenylalanine ammonia-lyase, POX or POD: peroxidase, PPO: polyphenol oxidase, RDM: root DM, RFW: root FW, ROS: Reactive oxygen species, RWC: relative water content, SDM: shoot DM, SFW: shoot FW, SOD: superoxide dismutase, AMF: arbuscular mycorrhizal fungi.

### 3.3. Metaorganism as a Strategy to Improve Phytoremediation

Recently, attention has been devoted to the plant and microbiome interactions as the “metaorganism approach” considering the close relationships between plants and microorganisms (symbiotic, non-symbiotic-rhizoephytic, and/or endophytic), which support tolerance to heavy metal stress and enhance the success of the phytoremediation process [159,160,161]. The metaorganism approach gathers the techniques related to (i) plant host selection, (ii) interference on root exudates, (iii) modification of the driving forces in the plant–microbiome interaction, and (iv) using meta-omics to obtain adequate information on the isolation and the application of microorganisms [160,161,162,163]. Plants are commonly pre-selected for traits, such as exceptional contaminant tolerance, high biomass, rapid growth, extensive root network, easy cultivation, repulsion to herbivores, and storage of HMs at high levels [20,35,56]. Plant selection is far more important than previously thought, owing to the association between plant phylogeny and microbial taxa, which can be altered under contamination. Bell et al. [164] demonstrated that total zinc accumulation in three willow cultivars was better explained by the fungal community structure, and concluded that the microbiome has the greatest impact on plant function and Zn extraction. In comparison to native plant species, non-native ones have been shown to form less beneficial associations with soil microbiome, which may reduce phytoremediation activity [165]. In this respect, other studies have established that the plant microbiome is important for plant growth, nutrition, and health, and directly and/or indirectly affects the composition, biomass, and functioning of plant communities, and should be considered in plant selection [166,167]. A better understanding of highly intimate plant–microbiome relationships could better predict potential positive or negative interactions. Rhizo-engineering is another technique that could ameliorate the phytoremediation process since root exudates play an important role in selecting and shaping rhizosphere microbiota, and there has been a major interest in changing the quality and quantity of root exudates via plant breeding and genetic modification to selectively stimulate specific microbial colonization [168]. Root exudates are composed of diverse compounds, which act as chemoattractant signals and/or carbon and nitrogen sources for microbes, thereby creating a unique environment in the rhizosphere. The rhizomicrobiome composition differs according to root exudate composition, as it changes along with the root system due to plant genotype and development stages and hence the phytoremediation process [140,160]. Identifying, understanding, and modifying the driving forces between the host and its microbiome are important to optimize the metaorganism. Studies suggest that a contaminated rhizosphere is more selective than a non-contaminated rhizosphere based on metatranscriptomic data [169,170,171,172]. Yergeau et al. [173] demonstrated that the combined selective pressure of contaminants and rhizosphere resulted in a higher expression of genes related to competition (antibiotic resistance and biofilm formation) in the contaminated rhizosphere, and thus genes related to phytoremediation were generally more expressed. Accordingly, *ars*C and *ere*A genes coding for resistance mechanisms to arsenic and macrolides, respectively, are the heaviest metal resistance genes (MRGs) and antibiotic resistance genes (ARGs) in a copper tailings dam area in northern China. The abundance of MRGs is positively correlated with Cd concentration, indicating the importance of Cd in the selection of MRGs [174]. In addition, phytoremediation systems can be optimized by focusing some energies at the metaorganisms omics level instead of solely focusing on plants and microbes [175]. Characterizing the plant–microbe metaorganism is considered the most powerful omics technology in phytoremediation since it leads to a new understanding of how integrated biological communities interact to adapt to contaminant stress and improve remediation [175]. Current novel omics approaches (especially next-generation sequencing technologies) combined with new bioinformatics techniques will provide intuitions on the microbe’s community and the ecology of the entire meta-communities, offering a wide range of opportunities for optimization and a better understanding of metaorganism-based approaches, that can be used to study the hyperaccumulator plant–microbial rhizobiome interactions, to maximize plant growth, for appropriate microbial community assembly, and ultimately, to enhance the phytoremediation process [175,176]. However, to advance further in the phytoremediation process, coordinated efforts from microbiologists, plant physiologists, molecular biologists, ecologists, soil scientists, environmental engineers, chemists, agronomists, and government regulators are needed [177,178,179,180].

### 3.4. Microbial Remediation: Heavy-Metal Remediation by Microorganisms

**Figure 4:**
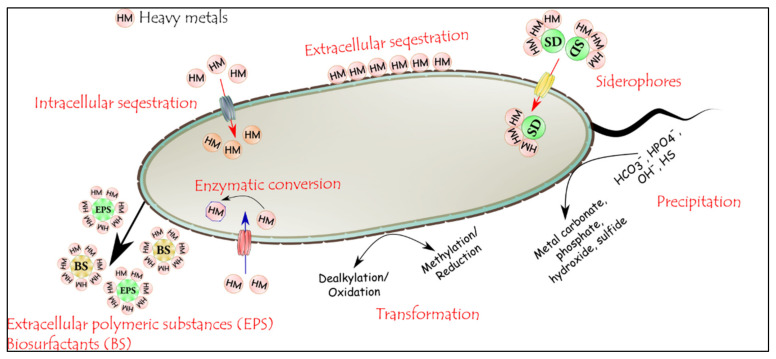
Schematic representation of various mechanisms involved in microbial remediation of HM-contaminated soil. HMs: Heavy metals; SD: Siderophores; EPS: Extracellular polymeric substances; BS: Biosurfactants.

Microbial remediation is a strategy that exploits the properties of endogenous (native) or exogenous microorganisms’ application to transform environmental contaminants into harmless forms [20,70,181]. The soil microbial community is widespread and highly diverse, with unlimited, undiscovered, and unexplored potential for HMs’ remediation. The most frequent microorganisms used for HMs’ remediation in contaminated soils are bacteria and fungi, although yeast and microalgae are often beneficial [10,12,13,19]. The microbial remediation for soil polluted with HMs depends on the active metabolizing capacities of microorganisms since metals do not readily undergo either chemically or biologically induced degradation [70,181]. Notwithstanding, metal-tolerant microorganisms can depollute HMs via several techniques (Figure 4), including extracellular complexation, intracellular accumulation, redox reactions, volatilization, and precipitation [9,70]. Biosorption is the most important mechanism of microbial remediation. Extracellular materials cause the immobilization of HMs by binding to anionic functional groups on the cell surface [20,181,182]. Numerous microorganisms were reported owing to their capacities for HMs’ remediation, such as *Nostoc linckia* [13], *B. megaterium*, *Rhizopus stolonifer* [183], *B. subtilis*, *Lecythophora* sp. [19], and *Saccharomyces cerevisiae* [10]. *Sporosarcina ginsengisoli* was found to decrease the exchangeable arsenic fraction of soil by producing a significant amount of urease (a precipitating calcite enzyme) [184]. Imam et al. [185] demonstrated that *B. subtilis* absorbed 76% of Cd^2+^ and 30% of Hg^2+^ from contaminated soil, while *S. cerevisiae* was able to take 70% of Cd^2+^ and 20% of Hg^2+^. Co-application of microorganisms has proved to be successful in remediation programs rather than single inoculum since shortfalls in one of them may be compensated. Kang et al. [186] confirmed that the bacterial mixtures (*Viridibacillus arenosi*, *Sporosarcina soli*, and *Enterobacter cloacae*) exhibited greater resistance and a considerably higher HM bioremediation capacity than single-strain cultures. It is worth mentioning that microorganism selection should be carried out based on the mechanisms involved to restore soil health. To improve microbial remediation, diverse approaches could be employed depending on the type of metals and the contaminated environment.

#### 3.4.1. Different Microbial Remediation Techniques Involving Removal and Containment of Contaminants

##### Bio-Stimulation

Bio-stimulation makes the soil auspicious for indigenous microorganisms by adding nutrients to the contaminated sites [70,187,188]. The addition of nutrients as manure, organic, and/or mineral amendments serves as a C source for the telluric microorganisms [187,188]. Consequently, growth, abundance, and microorganisms’ activities involved in the remediation, and thus bioremediation’s rate and efficiency, would be amplified [70,187,188]. Compost and biochar are currently the most common organic materials being exploited for their potential in the bio-stimulation processes. Raklami et al. [31] reported a significant decrease in the HMs’ fraction when the polluted soil was amended with 10% compost. Compost is a rich amendment with organic compounds (such as alcoholic, phenolic, and exopolysaccharides) that could retain HMs and convert them to their less-soluble form, and thus reduce their mobility [31,70,189]. Since the characteristics of organic amendment vary widely depending on the feedstock used for their production, their effects on the remediation and bio-stimulation processes could also differ. It is also important to note that the concentration of the amendment could have a considerable effect on the remediation process.

##### Bio-Augmentation

Bio-augmentation consists of the addition of pre-adapted, competent strains or consortia of microorganisms, the introduction of genetically modified microorganisms, or the addition of bioremediation-relevant gene packages in a vector to be transferred by conjugation into indigenous microorganisms [70,190]. The rationale for this approach is that the metabolic capacities of the indigenous microbial community may not be capable of remediating the HM-contaminated soils, or they may not tolerate the HM stress [188]. Another condition under which bio-augmentation may be considered is when the indigenous microbiota is low due to the recent exposure to HMs [70,190]. To succeed in this approach, the inoculum must be tolerant to HMs, maintain genetic constancy, endure in the receptive environment upon introduction, and successfully compete with the indigenous microbiota. Several studies have demonstrated that bio-augmentation significantly reduced HMs’ concentration in polluted soils [70,77,191]. Fauziah et al. [191] revealed that remediation via bio-augmentation with *Bacillus* sp., *Lysinibacillus* sp., or *Rhodococcus* sp. reduced the metal concentration of the HM-induced contaminated soils.

##### Engineered Microbial Remediation

Generally, autochthonous microbial strains are less tolerant/less potent to eliminate and remediate HM-polluted soils; therefore, bio-augmentation by genetically engineered microbial strains can be adopted [192,193]. Engineered microbial remediation is an emerging technology that has received more scientists’ attention as an efficient approach to restoring HM-contaminated soils [7,194]. These genetic adjustments encompass the insertion of the desired gene(s) in a single microorganism to improve metabolic pathways involved in remediation features. For example, the study of genes responsible for HMs’ accumulation can help to transfer this trait to other beneficial microbes [8,195]. Genetically engineered microbes have acted as robust bioremediation tools with significant capacities to remediate HM-contaminated soils. The commonly used genetically engineered microorganisms employed are: *Ralstonia eutropha*, *B. subtilis*, *Escherichia coli*, *P. putida*, and *Sphingomonas desiccabilis* [196,197,198]. Zhu et al. [197] reported that genetically engineered *E. coli* could be used to efficiently treat Cd and Pb environmental pollution. The *E. coli* cells were genetically engineered by introducing a de novo synthetic heavy-metal-capturing gene (encoding a protein *SynHMB* containing a six-histidine tag, two cysteine-rich peptides, and a metallothionein sequence) and a synthetic type VI secretory system (*T6SS*) cluster of *P. putida*, endowing the synthetic cells (*SynEc2*) with a high ability to display the heavy-metal-capturing *SynHMB* on the cell surface. Owing to the surface exposure of the six-histidine tag on the synthetic bacteria and carboxyl groups on the modified magnetic nanoparticles (MNPs), the co-assembled synthetic bacterial cells and MNPs captured these heavy metals with high removal efficiency (>90% even at 50 mg/L of Cd^2+^ and 50 mg/L of Pb^2+^) and were conveniently recycled by artificial magnetic fields. Wang et al. [199] demonstrated that the transcriptional activation mechanism of metalloprotein *MerR* in *E. coli* was more efficient than the most current biosystems with limited adsorption capacities. The engineered bioremediation system could continuously reduce mercury contamination in wide concentrations by transforming highly toxic Hg^2+^ to volatile and much less deleterious Hg^0^ with extraordinary selectivity.

#### 3.4.2. Mechanism of Heavy Metal Remediation by Microorganisms

Microorganisms are essential in the remediation of HM-contaminated soils as they use many mechanisms to endure and cope with metal toxicity. Such mechanisms include extracellular/intracellular sequestration, production of metal chelators, precipitation, enzymatic detoxification, and volatilization [7,9] (Figure 4). Single or multiple detoxification mechanisms could be used by any microbes to remediate contaminated media.

Extracellular sequestration accumulates metal ions by cellular components in the periplasm or complexation of metal ions as insoluble compounds [200]. This strategy is considered a “pre-defense” strategy as it occurs outside the bacterial cell [201]. Microorganisms possess a negative charge in their cell wall because of anionic structures that facilitate microbes’ metal-binding through different interactions, including covalent bonding, ionic interactions, or van der Wall forces of attraction, and consequently, the remediation of contaminated media (Figure 4). These harmful sites are the hydroxyl, alcohol, phosphoryl, amine, carboxyl, ester, sulfhydryl, sulfonate, thioether, and thiol groups [200]. Microbial communities can also produce extracellular polymeric substances (EPS) such as polysaccharides, glycoproteins, lipopolysaccharides, and soluble peptides [9,202]. EPS are constituted by nucleic acids, lipids, proteins, and complex carbohydrates, and possess a substantial quantity of metal-binding sites (e.g., carboxyl, hydroxyl, amino, sulfhydryl, and phosphate) that enable the stabilization of HMs through biosorption [9,202,203]. For instance, Dobrowolski et al. [17] demonstrated that the EPS obtained from bacterial strains *Rhodococcus opacus* and *Rhodococcus rhodochrous* displayed very high adsorption affinity toward Cu^2+^ and Pb^2+^, Co^2+^, and Cr^6+^, and they could be a successful strategy to remove HMs. Additionally, some microorganisms have the ability to remediate HM-polluted soil through the production of various chelating agents, such as siderophores, glomalin, and biosurfactants, which play a crucial role in microorganisms’ tolerance against HM toxicity and thus could be utilized as an essential microbial remediation tool [9,35] (Figure 4). Siderophores such as hydroxamate are organic chelating ligands, excreted by bacteria and fungi, can bind the HMs (besides iron), including copper, zinc, and nickel, and can protect microorganisms from HM toxicity [204,205]. Similarly, glomalin, a glycoprotein copiously produced by almost all AMF, can bind HMs and form a complex that cannot be absorbed by the living cells, reducing the impact of toxic metals on other soil microorganisms [101,206]. González-Chávez et al. [206] demonstrated that the glomalin extracted from two HM-polluted soils contained 1.6–4.3 mg Cu, 0.02–0.08 mg Cd, and 0.62–1.12 mg Pb/g glomalin. The biosurfactants (multifunctional, amphiphilic, surface-active biomolecules) are distinguished products that have been successfully utilized in the remediation of toxic HMs [12,207] (Figure 4). Biosurfactants were widely used, as potential candidates, in the bioremediation process for their ionic nature, small size, low toxicity, multi-functionality, surface activity, and environmental compatibility [207,208,209]. Numerous microorganisms, such as *P. aeruginosa* [12], *B. cereus* [210], *Bacillus* sp. [209], *Candida bombicola* [211], *Citrobacter freundii* [212], and *C. freundii* [211], have been documented as potential biosurfactant producers (such as rhamnolipid, lipopeptide, and sophorolipid), demonstrating remarkable HMs’ removal (e.g., As, Cd, Zn, Pb, Cr, Hg, Mn, and Cu). Microbial remediation can also occur via the production of hydrogen sulfide [187,213,214] (Figure 4). It has been demonstrated that sulfate-reducing bacteria excrete a large amount of hydrogen sulfide in the extracellular environment and induce the precipitation of HMs, and thus the remediation of contaminated soils [215,216]. *Desulfovibrio desulfuricans*, a sulfate-reducing bacterium, can transform sulfate to hydrogen sulfate, which ultimately precipitates HMs (e.g., Cd and Zn) [215].

Similar to extracellular sequestration, intracellular sequestration is also another beneficial process referring to a slow metabolic-dependent removal mechanism, where the sequestration of HMs takes place inside the cell [9,217] (Figure 4). In the intracellular sequestration process, HMs are transported across the cell wall and enter the cell cytoplasm, where they are sequestered and prevented from reaching toxic levels [3,200,218]. HMs such as cadmium, zinc, copper, nickel, lead, and chromium are sequestered inside the microorganism cell [32,217,218]. These metals are typically shifted inside the cell by ion pumps, ion channels, endocytosis, and lipid permeation [3,219] (Figure 4). During intracellular sequestration, the concentration of the internalized heavy-metal ions is regulated by metal homeostasis, which involves an inactive complexation with high-affinity ligands to avoid the toxicity of HMs [219]. Inactive complexation, or bio-precipitation, may result from the excretion of special sulfide, cytosolic polyphosphates, and cysteine-rich proteins, such as metallothionein, glutathione, and phytochelatins, which form insoluble metal precipitates [24,220]. Some studies have demonstrated that the expression of metallothionein in bacterial cells induced a remarkable capacity for the removal and remediation of HMs [221,222]. Genetically engineered *E. coli* expressing four rice metallothioneins confers enhanced mercury tolerance, metal binding, and sequestration [221]. Previous studies have elucidated the critical role of glutathione, as an alternative chelator, in HMs’ intracellular sequestration [9,223]. As a result of the increasing exposition of As, the ectomycorrhizal fungus *Hebeloma cylindrosporum* accumulated As intracellularly and thus induced the glutathione biosynthesis pathway [224]. In the same way, Rehman and Anjum [223] have also emphasized the role of glutathione as a detoxifying agent in *Candida tropicalis* in response to Cd stress. In response to HM exposure, some microorganisms produce phytochelatins to cope with their toxicity. Phytochelatins are metal-binding, cysteine-rich peptides consisting of three amino acids: glutamate, cysteine, and glycine, and the sulfhydryl group in the cysteine molecule is responsible for metal sequestration [24,220]. In an attempt to increase microbial remediation, the phytochelatins synthase gene (SpPCS) from *Schizosaccharomyces pombe* was cloned and expressed in *P. putida* KT2440. The recombined strain KT2440-SpPCS exhibited enhanced Cd, Ag, and Hg resistance and Cd accumulation [225].

Microbial communities have demonstrated their transforming capabilities by changing HMs to a less-toxic form, which has played an essential role in the microbial remediation of HMs in polluted soils [22,226] (Figure 4). Microbial transformations of HMs include oxidation, reduction, alkylation, and methylation. Numerous HMs exist in more than one chemical oxidation form [70,182]. Theoretically, the toxicity and bioavailability of As largely depend on the chemical form, and As^3+^ is considerably more mobile and toxic than As^5+^ (As^3+^ is 100× more toxic than As^5+^) [182,227]. As^3+^ oxidation capacity has been seen in many bacterial strains, including *P. aeruginosa*, *P. resinovorans*, *Kocuria palustris*, *P. alcaligenes, Vogesella indigofera*, *Micrococcus* sp., and *Acinetobacter* sp. [182,228], which is essential from the point of view of metal immobilization and bioremediation of contaminated areas. In addition, microorganisms can remediate several HMs by reducing them to a lower redox form [182,200] (Figure 4). This process is reversed as compared to the oxidation process. HMs which have many oxidation states remained insoluble in their reduced state. The microbial enzymatic reduction proved helpful in removing and remediating such elements from the solution [229,230]. Many beneficial microorganisms have been characterized by anaerobic or aerobic reduction of chromium and mercury as an effective means of Cr and Hg detoxification. *Ochrobactrum intermedium*, an indigenous chromium-reducing bacterial strain isolated from a tannery waste, can be an effective means of chromium detoxification (leads to the reduction of highly toxic chromium (VI) to less-toxic chromium (III)) under a wide range of environmental conditions [231]. *Shewanella oneidensis*, *Geobacter sulfurreducens*, and *Geobacter metallireducens* can reduce ionic mercury to elemental mercury and decrease its toxicity [232]. In addition to oxidation and reduction reactions, microorganisms can bioremediate contaminated soils by converting HMs (e.g., Pb, Hg, Se, As, Tn, and Sn) to methyl derivatives that are subsequently removed by volatilization [55,182]. It has been demonstrated that numerous microorganisms can transfer a methyl group to the metals, which results in methylated derivatives that differ in volatility, solubility, and toxicity [233,234,235]. For instance, Hu et al. [236] have demonstrated that *D. desulphuricans* ND132 methylates elemental mercury. Likewise, *Penicillium* sp. and *Aspergillus* sp. could volatilize arsenic mainly as trimethyl arsine, followed by mono- and di-methyl arsine [237].

Table 3 presents a list of microorganisms, their role in bioremediation, and their removal efficiency.

**Table 3:** List of some microorganisms used for microbial remediation.

Group	Bioremediation	Metal	Metal Concentration (mg/L)	Remediation Efficiency (%)	Mechanism	Reference
Bacteria	*P. aeruginosa*	As, Cd, Zn	182, 20, 983	53, 90, 80	Biosurfactant production	[12]
	*R. opacus*	Pb, Cd, Ni, Co, Cr	100, 100, 250, 200, 100	n/s	Adsorption in exopolysaccharides	[17]
	*R. rhodochrous*	Pb, Cd, Ni, Co, Cr	100, 250, 250, 150, 150	n/s	Adsorption in exopolysaccharides	[17]
	*B. cereeus*	Cr, Fe, Mn, Ni, Cu, Cd, Zn	100, 100, 50, 50, 30, 30, 50	82, 92, 97, 43, 25, 31, 36	Reduction (for Cr)	[18]
	*S. ginsengisoli*	As	500	98	Precipitation	[184]
	*B. subtilis*	Hg, Cd	500	30, 76	Biosrption	[185]
	*B. cereus*	Pb, Cd, Cr	100	69, 54, 43	Biosurfactant production	[210]
	*Bacillus* sp.	Pb, Hg, Mn, Cd	1000	76, 98, 90, 100	Biosurfactant production	[209]
	*C. freundii*	Al, Cd, Cu, Fe, Pb, Mn, Zn	n/s	87, 40, 19, 34, 57, 25, 49	Biosurfactant production	[212]
	*D. desulfuricans*	Cd, Ni, Cr	100	100, 98, 74	Sulfate-reduction	[215]
	*Ensifer adhaerens*	Cr, Cu, Cd, Ni, Zn, Pb	150	80, 81, 80, 82, 80, 80	Bioaccumulation and biosorption	[218]
	*Acinetobacter* sp.	Cr	16	87	Reduction	[238]
	*Alcaligenes faecalis*	Cd	100	70	Adsorption and/or precipitation	[239]
	*Bacillus pumilus*	Pb	100	88	Adsorption and/or precipitation	[239]
	*Brevibacterium iodinium*	Pb	100	87	Adsorption and/or precipitation	[239]
	*P. aeruginosa*	Cd, Pb	100	76	Adsorption and/or precipitation	[239]
	*B. cereus*	Pb	100	79.26	Bioaccumulation and biosorption	[240]
	*Bacillus circulans*	Cr	1110	71	Reduction	[241]
	*Bacillus firmus*	Pb, Cu, Zn	1000	98, 75, 62	Adsorption in exopolysaccharides	[242]
	*B. licheniformis*	Hg	100	73	-	[243]
	*B. subtilis*	Cr	570	100	Reduction	[244]
	*P. aeruginosa*	Cr	570	100	Reduction	[244]
	*Bacillus xiamenensis*	Pb	100	99.19	Bioaccumulation and biosorption	[245]
	*Cellulosimicrobium* sp.	Cr	300	63	Reduction	[246]
	*D. desulfuricans*	Cr, Cu, Ni	200	56, 79, 90	-	[247]
	*Micrococcus* sp.	Cr, Ni	100, 50	90, 55	Biosrption	[248]
	*P. aeruginosa*	Cd, Pb	435, 905	92, 88	Biosurfactant production	[249]
	*Sporosarcina saromensis*	Cr	100	100	Reduction	[250]
	*Streptomyces* sp.	Zn	65.38, 32.69	36 or 43	Bioaccumulation or biosorption	[251]
Yeast	*S. cerevisiae*	Pb, Cd	25, 80	71, 77	Biosorption	[10]
	*S. cerevisiae*	Hg, Cd	500	19, 70	Biosorption	[185]
	*C. bombicola*	Cr, Pb, Zn, Cu, Cd	70	23, 10, 7, 5, 16	Biosurfactant production	[211]
	*C. tropicalis*	Cd	100	78	Biosorption	[223]
	*Candida parapsilosis*	Hg	100	80	-	[243]
	*S. cerevisiae*	Cr	570	96	Reduction	[244]
	*Candida sphaerica*	Fe, Zn, Pb	1877, 1470, 3038	89, 87, 70	Biosurfactant production	[252]
	*Cryptococcus* sp.	Zn	100	85	Biosurfactant production	[253]
	*Rhodotorula mucilaginosa*	Cr	200	27	Reduction	[254]
Fungi	*A. niger*	Cd, Cr	0.6, 0.4	79, 48	Bioaccumulation	[11]
	*Aspergillus fumigatus*	Cd, Cr	0.6, 0.4	76, 35	Bioaccumulation	[11]
	*Penicillium rubens*	Cd, Cr	0.6, 0.4	75, 35	Bioaccumulation	[11]
	*Lecythophora* sp.	As	10	32	Reduction and volatilization	[19]
	*S. chinense*	Cd, Pb	400, 2000	88, 58	Biosorption	[150]
	*Aspergillus* sp.	Cr, Ni	100, 50	92, 9	Biosorption	[248]
	*Aspergillus flavus*	Cd, Cu, Fe, Mn, Pb, Zn	1000	87, 83, 96, 92, 87, 70	Biosorption	[255]
	*Aspergillus gracilis*	Cd, Cu, Fe, Mn, Pb, Zn	1000	66, 57, 90, 77, 82, 7	Biosorption	[255]
	*Aspergillus penicillioides*	Cd, Cu, Fe, Mn, Pb, Zn	1000	53, 32, 90, 69, 77, 84	Biosorption	[255]
	*Aspergillus restrictus*	Cd, Cu, Fe, Mn, Pb, Zn	1000	61,77, 64, 72, 44, 87	Biosorption	[255]
	*Sterigmatomyces halophilus*	Cd, Cu, Fe, Mn, Pb, Zn	1000	95, 90, 77, 89, 57, 93	Biosorption	[255]
	*A. niger*	Ni	30	70.3	Biosorption	[256]
	*Aspergillus versicolor*	Cr, Ni, Cu	50	100, 30, 29	Bioaccumulation	[257]
	*Phanerochaete chrysosporium*	Cd, Ni	25, 16	96, 89	Bioaccumulation	[258]
Cyanobacteria	*N. linckia*	Cr, Fe, Ni, Zn, Cu	2.5, 2, 0.5, 0.5, 0.5	n/s	Bioaccumulation	[13]
	*Chlorella pyrenoidosa*	Cu	5	83	Biosorption	[53]
	*Anabaena variabilis*	Pb	15	71.4	Biosorption and/or bioaccumulation	[259]
	*Nostoc muscorum*	Pb	15	97.8	Biosorption and/or bioaccumulation	[259]
	*Botryocossuss* sp.	Cr	5	94	Reduction and biosorption	[260]
	*C. pyrenoidosa*	Cd	1.5	45.45	Biosorption and bioaccumulation	[261]
	*Scenedesmus acutus*	Cd	1.5	57.14	Biosorption and bioaccumulation	[261]
	*Scenedesmus* sp.	Cr	10	93	Biosorption	[262]
	*Spirogyra* sp.	Cr, Cu, Fe, Mn, Se, Zn	5	98, 90, 100, 100, 98, 81	Biosorption and/or bioaccumulation	[263]
	*S. platensis*	Cr, Fe, Ni, Zn	10, 5, 2, 2	n/s	Bioaccumulation	[264]
	*Spirulina* sp.	Cr, Cu, Fe, Mn, Se, Zn	5	98, 81, 99, 100, 99, 79	Biosorption and/or bioaccumulation	[263]

n/s: Not specified.

#### 3.4.3. Soil Microbiota Evolution under HMs Contamination and Phytoremediation Approach

HMs have been known to disrupt ecosystem structure and functioning by disordering soil quality, substantial changes in microbial diversity, and biogeochemical cycles [265,266]. Invariably, all metals (single or in combination) created at least some change in the microbial community [169]. Microbial soil diversity is extremely rich, but in moderate HM exposure, the microbial diversity is reduced by more than 1000×, while during chronic contamination, it may account for only 1% of the initial community [5,170,171]. Several studies have demonstrated that reduced diversity is a commonly observed effect since HMs target specific pathways, resulting in disruption of definitive metabolic functions and selective inhibition, causing a decline in both numbers and diversity of the microbial community relying on those pathways and the proliferation of a few specific resistant groups [172]. For instance, Fajardo et al. [266] demonstrated that after 55 days, HM (Pb, Cd, and Zn) exposure had a strong effect on metabolic pathways, specifically, genetic information processing (transcription factors), metabolism (glycan biosynthesis and energy metabolism), and environmental processing (transporters and ABC transporters). Ecotoxicological tests showed that HMs induced a selective pressure on the soil microbiome, and the bacterial composition of the samples varied as the exposure time increased [266]. *Firmicutes* are the most resistant phylum, while the abundance of other bacterial phyla, such as *Proteobacteria*, *Actinobacteria*, *Verrucomicrobia*, and *Bacteroidetes*, decreased over time in the HM-spiked soil samples [266]. On the contrary, Li et al. [267] revealed that *Proteobacteria, Chloroflexi*, and *Acidobacteria* were the abundant phyla in the highly contaminated soils, and the abundance of *Proteobacteria* increased with the increase of HMs’ content, especially Cd, Ti, V, Cr, Co, Zn, As, Rb, Sr, Zr, Pb, and Bi. Similarly, Qiao et al. [268] found that in the farmland surrounding gold tailings (contaminated by HMs, mainly As, Pb, and Cd), *Actinobacteria* and *Proteobacteria* were the most abundant phyla in the bacterial community, followed by *Acidobacteria*, *Chloroflexi*, and *Gemmatimonadetes*. *Actinobacteria* content was reduced by increasing the level of the contamination, while the abundance of *Proteobacteria* was amplified. In this line, several other reports demonstrated that *Proteobacteria* tend to be the dominant bacteria in soil with long-term HM contamination as they exhibit better tolerance to HM pollution [269,270,271,272,273]. In other cases, microbial diversity may not be affected and remain constant, even under high exposure levels [274,275]. These results could be related to the strong resistance and resilience of the initial community and/or the capacity to acquire new resistant genes.

The remediation approaches also contribute to the substantial change in the microbial community. Huang et al. [276] demonstrated that the total concentration of HMs in two contaminated areas was diluted, and soil physicochemical characteristics were changed during the remediation approach of soil mixing. This change in soil has led to significant alterations of microbial diversity of the remediated areas. *Proteobacteria* and *Acidobacteria* were the phyla displaying a significant increase, while *Chloroflexi* was the only phylum that appeared to significantly reduce. Similarly, Wu et al. [277] revealed that the abundance of *Proteobacteria* and *Actinobacteria* was remarkably higher in revegetated tailings with *Lespedeza bicolor*, while *Chloroflexi* showed the opposite. After 13 years of phytoremediating an abandoned mine land in eastern China with white clover, ryegrass, and alfalfa, the soil microbial diversity was higher than the control [278]. Several additional studies highlighted the positive effect of the revegetation process and the phytoremediation approach in restoring the microbial diversity and activity in contaminated areas with HMs [279,280,281,282,283]. This positive effect could be related to roots exudates favoring microbial communities’ development.

#### 3.4.4. Microbial Remediation: Pitfalls, Drawbacks, and Future Application

Nowadays, microbial remediation has attracted scientists’ attention owing to its outstanding advantages, in contrast to the ‘traditional’ methods, as it exploits the natural ability of microorganisms to convert HMs into usually harmless forms [9,284]. Microbial remediation is simple, eco-friendly, sustainable, and relatively easy to implement in the waste treatment process for contaminated environments [285]. Additionally, this process can be carried out in situ, does not require excavation of the contaminated soil, often no residual treatment is required, and it proves to be cost-effective (vs. conventional clean-up treatments) [69,284,286]. The site disruption is minimal, and instantaneous management of soil and groundwater is possible [8,286]. Consequently, this strategy permits the chemical stabilization of HMs, reduces their water leaching and transport, and has fewer secondary effects on the surrounding environment, mainly when bio-stimulation, rather than bio-augmentation, is used [8,188]. Another advantage is that native microorganisms can be used in the clean-up process, for which no effort or manipulation in the environment for microbial growth is needed [20]. Microbial remediation is a natural process and is perceived positively by the public as a waste clean-up technique for contaminated soils [69].

However, there are still bottlenecks in its wide application. The process is slower and more time-consuming than other remedial methods [9]. The effectiveness of microbial remediation varies depending on the type of microorganism, its resistance, nature, level, and the synergetic toxicity of HMs (the process is precise) [9,181]. For instance, the effectiveness of this strategy depends on several factors, including the presence of microbial populations, suitable growth conditions, and appropriate levels of nutrients and pollutants [285]. Bioremediation is relatively sensitive to environmental factors (pH, humidity, temperature, presence of other ions and humic colloidal substances), and other living organisms and their competitors could play an essential role in the colonization of microorganisms of interest and the biofilm formation [9,181,285]. When these conditions are not satisfactory, the ability of the microbial population could be reduced [188,285]. A significant limitation of microbial remediation is that the HMs are concentrated or converted to less-toxic forms, but their presence is still in the soil [8]. Besides, the volatilization of some HMs causes atmospheric pollution, and hence controlling this process may be complex [287]. Indeed, hydrological and geochemical conditions may change over time, which could cause the remobilization of contaminants previously stabilized, and the risk of contaminants leaching into groundwater has to be tackled [288]. Another disadvantage faced by microbial remediation is monitoring ambiguity regarding the acceptable performance criteria of the level/definition of a ‘clean’ site, making the performance endpoint regulations uncertain and thus the evaluation of microbial remediation complex [286]. So far, four significant restrictions of using microbial remediation have been visualized, which hampers the spread of microbial strategies: (i) complications in extrapolating the technique from laboratory experiments and pilot-scale studies to full-scale field operations, (ii) insufficient understanding of the biological processes, the reaction involved to predict the output, and how it would react in the field, (iii) its management and anomalies in stimulating the microbe, and (iv) the struggle in ensuring proper contact or engagement between the microbe and HMs [182,286].

Research is required to advance and engineer microbial remediation strategies appropriate for sites with complex environmental stresses. The molecular mechanisms of heavy-metal detoxification need to be further expounded to enhance microorganisms’ accumulation of HMs. With an advanced understanding of the microbial remediation process and the mechanisms involved at the ecological and genetic levels, novel microbial remediation methods for bioremediation of HM-contaminated soils could be available.

### 3.5. Conclusions and Future Perspectives

Environmental pollution is a vital issue and is considered one of the biggest challenges of this century. Finding effective solutions to preserve the environment for the future generation is a time-demanding task. Environmental pollution, especially HM pollution, results in drastic effects on soil quality, fertility, loss of microbial biodiversity, and destruction of the vegetal cover. Scientists have developed several physicochemical strategies to remediate soils contaminated with HMs. Although these strategies are equitably widespread and suitable for removing HMs, they are challenging to apply, very expensive, non-specific, inefficient in a specific situation, and can significantly alter soil quality. As alternatives, new biological-based methods for the remediation of contaminated soils have been established. Biotechnological strategies, which exploit plants and microorganisms’ tolerance and properties (bacteria, microalgae, yeast, and fungi) to detoxify and stabilize HMs, have arisen as emerging and innovative technologies, and demonstrate increasing opportunities to restore HM-contaminated soils. Microbial remediation and phytoremediation are reliable, cost-effective, efficient, eco-feasible alternatives. Microorganisms and plants possess inherent mechanisms to tolerate toxic elements and remove metals from the environment. The microbes use various mechanisms, including extracellular/intracellular sequestration, metal chelators’ production, precipitation, enzymatic detoxification, and volatilization. The microbial remediation strategy could be coupled with phytoremediation methods (phytostabilization, phytoextraction, and phytovolatilization) for an effective reclamation. Engineered microbes and plants could add a layer to the bioremediation effectiveness that could surpass the limitations that each strategy suffers from.

A single strategy is neither effective nor sufficient for operational reclamation of HM-contaminated soils. The combination of different approaches—physical, chemical, and biological—is essential for highly effective and exhaustive phytoremediation in the future, and should be promoted. Research must focus on evaluating the effect of combining different microbes on phytoremediation efficiency, such as coupled microbial remediation with organic and inorganic chelating amendments. Besides, metagenomics approaches and microbial metabolic analysis need to be explored to select promising metal resistance and detoxification genes that could be de-regulated in other species to improve their specific performance. Metagenomics approaches should also focus on the evolution of microbial communities during the bioremediation process and determine the best strategies to enhance their survival because of the possible competitiveness with indigenous microbial populations and because survivability when released into the environment during the field trials is currently insufficient. Moreover, more genomic research is required to fully understand the metabolic pathways and the mechanisms involved in microbes and plants’ tolerance and detoxification of HMs.

## Data Availability

Not applicable.

## References

[1] Xu Y., Dai S., Meng K., Wang Y., Ren W., Zhao L., Christie P., Teng Y. (2018). Occurrence and risk assessment of potentially toxic elements and typical organic pollutants in contaminated rural soils. Sci. Total Environ..

[2] Guo J.J., Huang X.P., Xiang L., Wang Y.Z., Li Y.W., Li H., Cai Q.Y., Mo C.H., Wong M.H. (2020). Source, migration and toxicology of microplastics in soil. Environ. Int..

[3] Rajendran S., Priya T.A.K., Khoo K.S., Hoang T.K.A., Ng H.S., Munawaroh H.S.H., Karaman C., Orooji Y., Show P.L. (2021). A Critical review on various remediation approaches for heavy metal contaminants removal from contaminated soils. Chemosphere.

[4] Emenike C.U., Jayanthi B., Agamuthu P., Fauziah S.H. (2018). Biotransformation and removal of heavy metals: A review of phytoremediation and microbial remediation assessment on contaminated soil. Environ. Rev..

[5] Tang J., Zhang J., Ren L., Zhou Y., Gao J., Luo L., Yang Y., Peng Q., Huang H., Chen A. (2019). Diagnosis of soil contamination using microbiological indices: A review on heavy metal pollution. J. Environ. Manag..

[6] El Alaoui A., Raklami A., Bechtaoui N., El Gharmali A., Ouhammou A., Imziln B., Achouak W., Pajuelo E., Oufdou K. (2021). Use of native plants and their associated bacteria rhizobiomes to remediate-restore Draa Sfar and Kettara mining sites, Morocco. Environ. Monit. Assess..

[7] Ojuederie O.B., Babalola O.O. (2017). Microbial and plant-assisted bioremediation of heavy metal polluted environments: A review. Int. J. Environ. Res. Public Health.

[8] Liu S., Yang B., Liang Y., Xiao Y., Fang J. (2020). Prospect of phytoremediation combined with other approaches for remediation of heavy metal-polluted soils. Environ. Sci. Pollut. Res..

[9] Yin K., Wang Q., Lv M., Chen L. (2019). Microorganism remediation strategies towards heavy metals. Chem. Eng. J..

[10] Hadiani M.R., Darani K.K., Rahimifard N., Younesi H. (2018). Biosorption of low concentration levels of lead (II) and cadmium (II) from aqueous solution by *Saccharomyces cerevisiae*: Response surface methodology. Biocatal. Agric. Biotechnol..

[11] Khan I., Aftab M., Shakir S.U., Ali M., Qayyum S., Rehman M.U., Haleem K.S., Touseef I. (2019). Mycoremediation of heavy metal (Cd and Cr)–polluted soil through indigenous metallotolerant fungal isolates. Environ. Monit. Assess..

[12] Lopes C.S.C., Teixeira D.B., Braz B.F., Santelli R.E., de Castilho L.V.A., Gomez J.G.C., Castro R.P.V., Seldin L., Freire D.M.G. (2021). Application of rhamnolipid surfactant for remediation of toxic metals of long- and short-term contamination sites. Int. J. Environ. Sci. Technol..

[13] Cepoi L., Zinicovscaia I., Valuta A., Codreanu L., Rudi L., Chiriac T., Yushin N., Grozdov D., Peshkova A. (2022). Bioremediation capacity of edaphic cyanobacteria *Nostoc linckia* for chromium in association with other heavy-metals-contaminated soils. Environments.

[14] Guarino F., Miranda A., Castiglione S., Cicatelli A. (2020). Arsenic phytovolatilization and epigenetic modifications in *Arundo donax* L. assisted by a PGPR consortium. Chemosphere.

[15] Yanitch A., Kadri H., Frenette-Dussault C., Joly S., Pitre F.E., Labrecque M. (2020). A four-year phytoremediation trial to decontaminate soil polluted by wood preservatives: Phytoextraction of Arsenic, Chromium, Copper, Dioxins and Furans. Int. J. Phytoremed..

[16] Gavrilescu M. (2022). Enhancing phytoremediation of soils polluted with heavy metals. Curr. Opin. Biotechnol..

[17] Dobrowolski R., Szcześ A., Czemierska M., Jarosz-Wikołazka A. (2017). Studies of Cadmium(II), Lead(II), Nickel(II), Cobalt(II) and Chromium(VI) sorption on extracellular polymeric substances produced by *Rhodococcus opacus* and *Rhodococcus rhodochrous*. Bioresour. Technol..

[18] Nayak A.K., Panda S.S., Basu A., Dhal N.K. (2018). Enhancement of toxic Cr (VI), Fe, and other heavy metals phytoremediation by the synergistic combination of native *Bacillus cereus* Strain and *Vetiveria zizanioides* L.. Int. J. Phytoremed..

[19] Chang J., Si G., Dong J., Yang Q., Shi Y., Chen Y., Zhou K., Chen J. (2021). Transcriptomic analyses reveal the pathways associated with the volatilization and resistance of Mercury(II) in the fungus *Lecythophora* sp. DC-F1. Sci. Total Environ..

[20] Ashraf S., Ali Q., Zahir Z.A., Ashraf S., Asghar H.N. (2019). Phytoremediation: Environmentally sustainable way for reclamation of heavy metal polluted soils. Ecotoxicol. Environ. Saf..

[21] Gustin M.S., Hou D., Tack F.M.G. (2021). The term “heavy metal(s)”: History, current debate, and future use. Sci. Total Environ..

[22] Pratush A., Kumar A., Hu Z. (2018). Adverse Effect of Heavy Metals (As, Pb, Hg, and Cr) on health and their bioremediation strategies: A review. Int. Microbiol..

[23] Asad S.A., Farooq M., Afzal A., West H. (2019). Integrated phytobial heavy metal remediation strategies for a sustainable clean environment—A review. Chemosphere.

[24] García-García J.D., Sánchez-Thomas R., Moreno-Sánchez R. (2016). Bio-recovery of non-essential heavy metals by intra- and extracellular mechanisms in free-living microorganisms. Biotechnol. Adv..

[25] Zwolak A., Sarzyńska M., Szpyrka E., Stawarczyk K. (2019). Sources of soil pollution by heavy metals and their accumulation in vegetables: A review. Water. Air. Soil Pollut..

[26] Dhaliwal S.S., Singh J., Taneja P.K., Mandal A. (2020). Remediation techniques for removal of heavy metals from the soil contaminated through different sources: A review. Environ. Sci. Pollut. Res..

[27] Kandziora-Ciupa M., Nadgórska-Socha A., Barczyk G. (2021). The influence of heavy metals on biological soil quality assessments in the *Vaccinium myrtillus* L. rhizosphere under different field conditions. Ecotoxicology.

[28] Sharma R., Singh N.S., Singh D.K. (2020). Impact of heavy metal contamination and seasonal variations on enzyme’s activity of Yamuna river soil in Delhi and NCR. Appl. Water Sci..

[29] Kumar S., Prasad S., Yadav K.K., Shrivastava M., Gupta N., Nagar S., Bach Q.V., Kamyab H., Khan S.A., Yadav S. (2019). Hazardous heavy metals contamination of vegetables and food chain: Role of sustainable remediation approaches—A review. Environ. Res..

[30] Mensah A.K., Marschner B., Antoniadis V., Stemn E., Shaheen S.M., Rinklebe J. (2021). Human health risk via soil ingestion of potentially toxic elements and remediation potential of native plants near an abandoned mine spoil in Ghana. Sci. Total Environ..

[31] Raklami A., Tahiri A., Bechtaoui N., Abdelhay E.G., Pajuelo E., Baslam M., Meddich A., Oufdou K. (2020). Restoring the plant productivity of heavy metal-contaminated soil using phosphate sludge, marble waste, and beneficial microorganisms. J. Environ. Sci..

[32] Li C., Quan Q., Gan Y., Dong J., Fang J., Wang L., Liu J. (2020). Effects of heavy metals on microbial communities in sediments and establishment of bioindicators based on microbial taxa and function for environmental monitoring and management. Sci. Total Environ..

[33] Bai X.T., Wang J., Dong H., Chen J.M., Ge Y. (2021). Relative importance of soil properties and heavy metals/metalloids to modulate microbial community and activity at a smelting site. J. Soils Sediments.

[34] Shuaib M., Azam N., Bahadur S., Romman M., Yu Q., Xuexiu C. (2021). Variation and succession of microbial communities under the conditions of persistent heavy metal and their survival mechanism. Microb. Pathog..

[35] Jacob J.M., Karthik C., Saratale R.G., Kumar S.S., Prabakar D., Kadirvelu K., Pugazhendhi A. (2018). Biological approaches to tackle heavy metal pollution: A survey of literature. J. Environ. Manag..

[36] Aponte H., Meli P., Butler B., Paolini J., Matus F., Merino C., Cornejo P., Kuzyakov Y. (2020). Meta-analysis of heavy metal effects on soil enzyme activities. Sci. Total Environ..

[37] AL-huqail A.A., El-bondkly A.M.A. (2021). Improvement of *Zea mays* L. Growth parameters under chromium and arsenic stress by the heavy metal-resistant *Streptomyces* sp. NRC21696. Int. J. Environ. Sci. Technol..

[38] Raklami A., Oufdou K., Tahiri A.I., Mateos-Naranjo E., Navarro-Torre S., Rodríguez-Llorente I.D., Meddich A., Redondo-Gómez S., Pajuelo E. (2019). Safe cultivation of *Medicago sativa* in metal-polluted soils from semi-arid regions assisted by heat-and metallo-resistant PGPR. Microorganisms.

[39] Raklami A., Oubane M., Meddich A., Hafidi M., Marschner B., Heinze S., Oufdou K. (2021). Phytotoxicity and genotoxicity as a new approach to assess heavy metals effect on *Medicago sativa* L.: Role of metallo-resistant rhizobacteria. Environ. Technol. Innov..

[40] Tauqeer H.M., Ali S., Rizwan M., Ali Q., Saeed R., Iftikhar U., Ahmad R., Farid M., Abbasi G.H. (2016). Phytoremediation of heavy metals by *Alternanthera bettzickiana*: Growth and physiological response. Ecotoxicol. Environ. Saf..

[41] El-Mahdy O.M., Mohamed H.I., Mogazy A.M. (2021). Biosorption effect of *Aspergillus niger* and *Penicillium chrysosporium* for Cd- and Pb-contaminated soil and their physiological effects on *Vicia faba* L.. Environ. Sci. Pollut. Res..

[42] Wang X., Fang L., Beiyuan J., Cui Y., Peng Q., Zhu S., Wang M., Zhang X. (2021). Improvement of alfalfa resistance against Cd stress through rhizobia and arbuscular mycorrhiza fungi co-inoculation in cd-contaminated soil. Environ. Pollut..

[43] Jia Z., Li S., Wang L. (2018). Assessment of soil heavy metals for eco-environment and human health in a rapidly urbanization area of the upper Yangtze Basin. Sci. Rep..

[44] Edokpayi J.N., Enitan A.M., Mutileni N., Odiyo J.O. (2018). Evaluation of water quality and human risk assessment due to heavy metals in groundwater around Muledane area of Vhembe district, Limpopo province. S. Afr. Chem. Cent. J..

[45] Ravindra K., Mor S. (2019). Distribution and health risk assessment of arsenic and selected heavy metals in groundwater of Chandigarh, India. Environ. Pollut..

[46] Njoga E.O., Ezenduka E.V., Ogbodo C.G., Ogbonna C.U., Jaja I.F., Ofomatah A.C., Okpala C.O.R. (2021). Detection, Distribution and health risk assessment of toxic heavy metals/metalloids, arsenic, cadmium, and lead in goat carcasses processed for human consumption in south-eastern Nigeria. Foods.

[47] WHO (World Health Organization) (2017). Evolution of Who Air Quality Guidelines.

[48] Lai H.Y., Hseu Z.Y., Chen T.C., Chen B.C., Guo H.Y., Chen Z.S. (2010). Health risk-based assessment and management of heavy metals-contaminated soil sites in Taiwan. Int. J. Environ. Res. Public Health.

[49] Li C., Zhou K., Qin W., Tian C., Qi M., Yan X., Han W. (2019). A Review on heavy metals contamination in soil: Effects, sources, and remediation techniques. Soil Sediment Contam..

[50] Xia X., Lin S., Zhao J., Zhang W., Lin K., Lu Q., Zhou B. (2018). Toxic responses of microorganisms to nickel exposure in farmland soil in the presence of earthworm (*Eisenia fetida*). Chemosphere.

[51] Roychowdhury A., Datta R., Sarkar D. (2018). Heavy metal pollution and remediation. Green Chemistry.

[52] Mosa K.A., Saadoun I., Kumar K., Helmy M., Dhankher O.P. (2016). Potential biotechnological strategies for the cleanup of heavy metals and metalloids. Front. Plant Sci..

[53] Moreira V.R., Lebron Y.A.R., Freire S.J., Santos L.V.S., Palladino F., Jacob R.S. (2019). Biosorption of copper ions from aqueous solution using *Chlorella pyrenoidosa*: Optimization, equilibrium and kinetics studies. Microchem. J..

[54] McGrath S.P., Zhao F.J., Lombi E. (2001). Plant and rhizosphere processes involved in phytoremediation of metal-contaminated soils. Plant Soil.

[55] Ullah A., Heng S., Farooq M., Munis H., Fahad S., Yang X. (2015). Phytoremediation of heavy metals assisted by plant growth promoting (PGP) bacteria: A review. Environ. Exp. Bot..

[56] Oladoye P.O., Olowe O.M., Asemoloye M.D. (2022). Phytoremediation technology and food security impacts of heavy metal contaminated soils: A review of literature. Chemosphere.

[57] Antoniadis V., Levizou E., Shaheen S.M., Ok Y.S., Sebastian A., Baum C., Prasad M.N.V., Wenzel W.W., Rinklebe J. (2017). Trace elements in the soil-plant interface: Phytoavailability, translocation, and phytoremediation—A review. Earth-Sci. Rev..

[58] Raklami A., Meddich A., Pajuelo E., Marschner B., Heinze S., Oufdou K. (2022). Combined application of marble waste and beneficial microorganisms: Toward a cost-effective approach for restoration of heavy metals contaminated sites. Environ. Sci. Pollut. Res..

[59] DalCorso G., Fasani E., Manara A., Visioli G., Furini A. (2019). Heavy metal pollutions: State of the art and innovation in phytoremediation. Int. J. Mol. Sci..

[60] Ali H., Khan E., Sajad M.A. (2013). Phytoremediation of heavy metals-concepts and applications. Chemosphere.

[61] Kavanagh L., Keohane J., Cabellos G.G., Lloyd A., Cleary J. (2018). Induced plant accumulation of lithium. Geosciences.

[62] Mahar A., Wang P., Ali A., Awasthi M.K., Lahori A.H., Wang Q., Li R., Zhang Z. (2016). Challenges and opportunities in the phytoremediation of heavy metals contaminated soils: A review. Ecotoxicol. Environ. Saf..

[63] Raklami A., El Gharmali A., Rahou Y.A., Oufdou K. (2020). Compost and mycorrhizae application as a technique to alleviate Cd and Zn stress in *Medicago sativa*. Int. J. Phytoremed..

[64] Fagorzi C., Checcucci A., DiCenzo G., Debiec-Andrzejewska K., Dziewit L., Pini F., Mengoni A. (2018). Harnessing rhizobia to improve heavy-metal phytoremediation by legumes. Genes.

[65] Nirola R., Megharaj M., Palanisami T., Aryal R., Venkateswarlu K., Naidu R. (2015). Evaluation of metal uptake factors of native trees colonizing an abandoned copper mine—A quest for phytostabilization. J. Sustain. Min..

[66] Jebara S.H., Saadani O., Fatnassi I.C., Chiboub M., Abdelkrim S., Jebara M. (2015). Inoculation of *Lens culinaris* with Pb-resistant bacteria shows potential for phytostabilization. Environ. Sci. Pollut. Res..

[67] Saadani O., Fatnassi I.C., Chiboub M., Abdelkrim S., Barhoumi F., Jebara M., Jebara S.H. (2016). Ecotoxicology and environmental safety in situ phytostabilisation capacity of three legumes and their associated plant growth promoting bacteria (PGPBs) in mine tailings of northern Tunisia. Ecotoxicol. Environ. Saf..

[68] Oleńska E., Imperato V., Małek W., Włostowski T., Wójcik M., Swiecicka I., Vangronsveld J., Thijs S. (2020). *Trifolium repens*-Associated bacteria as a potential tool to facilitate phytostabilization of zinc and lead polluted waste heaps. Plants.

[69] Singh J., Sharma D., Kumar G., Sharma N.R., Singh J., Sharma D., Kumar G., Sharma N.R. (2018). Bioremediation: An eco-sustainable approach for restoration of contaminated sites. Microbial Bioprospecting for Sustainable Development.

[70] Saha L., Tiwari J., Bauddh K., Ma Y. (2021). Recent developments in microbe–plant-based bioremediation for tackling heavy metal-polluted soils. Front. Microbiol..

[71] Sakakibara M., Watanabe A., Inoue M., Sano S., Kaise T. Phytoextraction and phytovolatilization of arsenic from as-contaminated soils by *Pteris vittata*. Proceedings of the Annual International Conference on Soils, Sediments Water and Energy.

[72] Tyagi B., Kumar N. (2021). Bioremediation: Principles and applications in environmental management. Bioremediation for Environmental Sustainability.

[73] Dolphen R., Thiravetyan P. (2015). Phytodegradation of ethanolamines by *Cyperus alternifolius*: Effect of molecular size. Int. J. Phytoremed..

[74] Chen F., Huber C., May R., Schröder P. (2016). Metabolism of Oxybenzone in a Hairy Root Culture: Perspectives for phytoremediation of a widely used sunscreen agent. J. Hazard. Mater..

[75] Rezania S., Taib S.M., Md Din M.F., Dahalan F.A., Kamyab H. (2016). Comprehensive review on phytotechnology: Heavy metals removal by diverse aquatic plants species from wastewater. J. Hazard. Mater..

[76] Hakeem K.R., Bhat R.A., Qadri H., Hakeem K.R., Bhat R.A., Qadri H. (2020). Phytoremediation of heavy metals: An eco-friendly and sustainable approach. Bioremediation and Biotechnology: Sustainable Approaches to Pollution Degradation.

[77] Kumar R., Mishra R.K., Mishra V., Qidwai A., Pandey A., Shukla S.K., Pandey M., Pathak A., Dikshit A. (2015). Detoxification and Tolerance of Heavy Metals in Plants.

[78] Sytar O., Ghosh S., Malinska H., Zivcak M., Brestic M. (2021). Physiological and molecular mechanisms of metal accumulation in hyperaccumulator plants. Physiol. Plant..

[79] Gupta D.K., Corpas F.J., Palma J.M., Gupta D.K., Corpas F.J., Palma J.M. (2013). Role of phytochelatins in heavy metal stress and detoxification mechanisms in plants. Heavy Metal Stress in Plants.

[80] Ali A., Guo D., Mahar A., Ma F., Li R., Shen F., Wang P., Zhang Z. (2017). *Streptomyces pactum* assisted phytoremediation in Zn/Pb smelter contaminated soil of feng county and its impact on enzymatic activities. Sci. Rep..

[81] Haydon M.J., Cobbett C.S. (2007). Transporters of ligands for essential metal ions in plants: Research review. New Phytol..

[82] Guo B.Y., Peng Z.L., Han F., Shan X.Q., Lin J.M. (2007). Study of low-molecular weight organic acids in maize roots under the stress of cadmium using capillary zone electrophoresis. J. Sep. Sci..

[83] Kanwal S., Bano A., Malik R.N. (2015). Effects of arbuscular mycorrhizal fungi on metals uptake, physiological and biochemical response of *Medicago sativa* L. with increasing Zn and Cd concentrations in Soil. Am. J. Plant Sci..

[84] Kushwaha A., Rani R., Kumar S., Gautam A. (2015). Heavy metal detoxification and tolerance mechanisms in plants: Implications for phytoremediation. Environ. Rev..

[85] Talukder P. (2021). Role of Metallothionein and Phytochelatin in Combating Abiotic Stress Imparted by Copper-Induced Toxicity in Brinjal (Solanum melongena).

[86] Yan A., Wang Y., Tan S.N., Mohd Yusof M.L., Ghosh S., Chen Z. (2020). Phytoremediation: A promising approach for revegetation of heavy metal-polluted land. Front. Plant Sci..

[87] Jian C., Yang Z., Su Y., Han F.X., Monts D.L., Steinberg V. (2011). Phytoremediation of heavy metal/metalloid-contaminated soils. Contaminated Soils: Environmental Impact, Disposal and Treatment.

[88] Fernández L.G., Fernández-pascual M., Javier F., Mañero G., Antonio J., García L., Ansari A.A., Gill S.S., Gill R., Lanza G., Newman L. (2015). Phytoremediation of contaminated waters to improve water quality. Phytoremediation.

[89] Liu W., Wang B., Wang Q., Hou J., Wu L., Wood J.L., Luo Y., Franks A.E. (2016). Characteristics of metal-tolerant plant growth-promoting yeast (*Cryptococcus* sp. NSE1) and its influence on Cd hyperaccumulator *Sedum plumbizincicola*. Environ. Sci. Pollut. Res..

[90] Anli M., Baslam M., Tahiri A., Raklami A., Symanczik S., Boutasknit A., Ait-El-Mokhtar M., Ben-Laouane R., Toubali S., Ait Rahou Y. (2020). Biofertilizers as strategies to improve photosynthetic apparatus, growth, and drought stress tolerance in the date palm. Front. Plant Sci..

[91] Ben-Laouane R., Ait-El-Mokhtar M., Anli M., Boutasknit A., Ait Rahou Y., Raklami A., Oufdou K., Wahbi S., Meddich A. (2021). Green compost combined with mycorrhizae and rhizobia: A strategy for improving alfalfa growth and yield under field conditions. Gesunde Pflanz..

[92] Navarro-Torre S., Mateos-Naranjo E., Caviedes M.A., Pajuelo E., Rodríguez-Llorente I.D. (2016). Isolation of plant-growth-promoting and metal-resistant cultivable bacteria from *Arthrocnemum macrostachyum* in the odiel marshes with potential use in phytoremediation. Mar. Pollut. Bull..

[93] Raklami A., Bechtaoui N., Tahiri A.I., Anli M., Meddich A., Oufdou K. (2019). Use of rhizobacteria and mycorrhizae consortium in the open field as a strategy for improving crop nutrition, productivity and soil fertility. Front. Microbiol..

[94] Nafis A., Raklami A., Bechtaoui N., El Khalloufi F., El Alaoui A., Glick B.R., Hafidi M., Kouisni L., Ouhdouch Y., Hassani L. (2019). Actinobacteria from extreme niches in morocco and their plant growth-promoting potentials. Diversity.

[95] Manoj S.R., Karthik C., Kadirvelu K., Arulselvi P.I., Shanmugasundaram T., Bruno B., Rajkumar M. (2020). Understanding the molecular mechanisms for the enhanced phytoremediation of heavy metals through plant growth promoting rhizobacteria: A review. J. Environ. Manag..

[96] Sharma P., Tripathi S., Chaturvedi P., Chaurasia D., Chandra R. (2021). Newly isolated *Bacillus* sp. PS-6 assisted phytoremediation of heavy metals using *Phragmites communis*: Potential application in wastewater treatment. Bioresour. Technol..

[97] Coninx L., Martinova V., Rineau F. (2017). Mycorrhiza-assisted phytoremediation. Adv. Bot. Res..

[98] Riaz M., Kamran M., Fang Y., Wang Q., Cao H., Yang G., Deng L., Wang Y., Zhou Y., Anastopoulos I. (2021). Arbuscular mycorrhizal fungi-induced mitigation of heavy metal phytotoxicity in metal contaminated soils: A critical review. J. Hazard. Mater..

[99] Liu L., Li J., Yue F., Yan X., Wang F., Bloszies S., Wang Y. (2018). Effects of arbuscular mycorrhizal inoculation and biochar amendment on maize growth, cadmium uptake and soil cadmium speciation in cd-contaminated soil. Chemosphere.

[100] Janoušková M., Pavlíková D., Vosátka M. (2006). Potential contribution of arbuscular mycorrhiza to cadmium immobilisation in soil. Chemosphere.

[101] Cabral L., Soares C.R.F.S., Giachini A.J., Siqueira J.O. (2015). Arbuscular mycorrhizal fungi in phytoremediation of contaminated areas by trace elements: Mechanisms and major benefits of their applications. World J. Microbiol. Biotechnol..

[102] Khalid M., Ur-rahman S., Hassani D., Hayat K., Zhou P., Hui N. (2021). Advances in fungal-assisted phytoremediation of heavy metals: A review. Pedosphere.

[103] Handa Y., Nishide H., Takeda N., Suzuki Y., Kawaguchi M., Saito K. (2015). RNA-Seq transcriptional profiling of an arbuscular mycorrhiza provides insights into regulated and coordinated gene expression in *Lotus japonicus* and *Rhizophagus irregularis*. Plant Cell Physiol..

[104] Das J., Sarkar P. (2018). Remediation of arsenic in mung bean (*Vigna radiata*) with growth enhancement by unique arsenic-resistant bacterium *Acinetobacter lwoffii*. Sci. Total Environ..

[105] Hu T., Chen A., Jiang Y., Sun C., Luo S., Shao J. (2022). Application of a newly recorded diazotrophic cyanobacterium in acidified and Cd contaminated paddy soil: Promotes rice yield and decreases Cd accumulation. Sci. Total Environ..

[106] Pan F., Meng Q., Luo S., Shen J., Chen B., Khan K.Y., Japenga J., Ma X., Yang X., Feng Y. (2017). Enhanced Cd extraction of oilseed rape (*Brassica napus*) by plant growth-promoting bacteria isolated from cd hyperaccumulator *Sedum alfredii* Hance. Int. J. Phytoremed..

[107] Visioli G., Vamerali T., Mattarozzi M., Dramis L., Sanangelantoni A.M. (2015). Combined endophytic inoculants enhance nickel phytoextraction from serpentine soil in the hyperaccumulator *Noccaea caerulescens*. Front. Plant Sci..

[108] Hassan T.U., Bano A., Naz I. (2017). Alleviation of heavy metals toxicity by the application of plant growth promoting rhizobacteria and effects on wheat grown in saline sodic field. Int. J. Phytoremed..

[109] Bruno L.B., Anbuganesan V., Karthik C., Tripti, Kumar A., Banu J.R., Freitas H., Rajkumar M. (2021). Enhanced phytoextraction of multi-metal contaminated soils under increased atmospheric temperature by bioaugmentation with plant growth promoting *Bacillus cereus*. J. Environ. Manag..

[110] Funes Pinter I., Salomon M.V., Berli F., Bottini R., Piccoli P. (2017). Characterization of the As(III) tolerance conferred by plant growth promoting rhizobacteria to in vitro-grown grapevine. Appl. Soil Ecol..

[111] Wang Q., Zhang W.J., He L.Y., Sheng X.F. (2018). Increased biomass and quality and reduced heavy metal accumulation of edible tissues of vegetables in the presence of Cd-tolerant and immobilizing *Bacillus megaterium* H3. Ecotoxicol. Environ. Saf..

[112] Mohammadzadeh A., Tavakoli M., Motesharezadeh B., Chaichi M.R. (2017). Effects of plant growth-promoting bacteria on the phytoremediation of cadmium-contaminated soil by sunflower. Arch. Agron. Soil Sci..

[113] Ahmad I., Akhtar M.J., Asghar H.N., Ghafoor U., Shahid M. (2016). Differential effects of plant growth-promoting rhizobacteria on maize growth and cadmium uptake. J. Plant Growth Regul..

[114] Motesharezadeh B., Kamal-poor S., Alikhani H., Zarei M., Azimi S. (2017). Investigating the effects of plant growth promoting bacteria and *Glomus mosseae* on cadmium phytoremediation by *Eucalyptus camaldulensis* L.. Pollution.

[115] Mousavi S.M., Motesharezadeh B., Hosseini H.M., Alikhani H., Zolfaghari A.A. (2018). Root-Induced Changes of Zn and Pb dynamics in the rhizosphere of sunflower with different plant growth promoting treatments in a heavily contaminated soil. Ecotoxicol. Environ. Saf..

[116] Płociniczak T., Sinkkonen A., Romantschuk M., Sułowicz S., Piotrowska-Seget Z. (2016). Rhizospheric bacterial strain *Brevibacterium casei* MH8a colonizes plant tissues and enhances Cd, Zn, Cu phytoextraction by white mustard. Front. Plant Sci..

[117] Haruma T., Yamaji K., Ogawa K., Masuya H., Sekine Y., Kozai N. (2019). Root-endophytic *Chaetomium cupreum* chemically enhances aluminium tolerance in *Miscanthus sinensis* via increasing the aluminium detoxicants, chlorogenic acid and oosporein. PLoS ONE.

[118] Awasthi S., Chauhan R., Dwivedi S., Srivastava S., Srivastava S., Tripathi R.D. (2018). A Consortium of alga (*Chlorella vulgaris*) and bacterium (*Pseudomonas putida*) for amelioration of arsenic toxicity in rice: A promising and feasible approach. Environ. Exp. Bot..

[119] Kaur J., Anand V., Srivastava S., Bist V., Tripathi P., Naseem M., Nand S., Anshu, Khare P., Srivastava P.K. (2020). Yeast strain *Debaryomyces hansenii* for amelioration of arsenic stress in rice. Ecotoxicol. Environ. Saf..

[120] Adeyemi N.O., Atayese M.O., Sakariyawo O.S., Azeez J.O., Abayomi Sobowale S.P., Olubode A., Mudathir R., Adebayo R., Adeoye S. (2021). Alleviation of heavy metal stress by arbuscular mycorrhizal symbiosis in *Glycine max* (L.) grown in copper, lead and zinc contaminated soils. Rhizosphere.

[121] Zhan F., Li B., Jiang M., Li T., He Y., Li Y., Wang Y. (2019). Effects of arbuscular mycorrhizal fungi on the growth and heavy metal accumulation of bermudagrass [*Cynodon dactylon* (L.) Pers.] grown in a lead–zinc mine wasteland. Int. J. Phytoremed..

[122] Zhang F., Liu M., Li Y., Che Y., Xiao Y. (2019). Effects of arbuscular mycorrhizal fungi, biochar and cadmium on the yield and element uptake of *Medicago sativa*. Sci. Total Environ..

[123] Liu H., Yuan M., Tan S., Yang X., Lan Z., Jiang Q., Ye Z., Jing Y. (2015). Enhancement of arbuscular mycorrhizal fungus (*Glomus versiforme*) on the growth and Cd uptake by Cd-hyperaccumulator *Solanum nigrum*. Appl. Soil Ecol..

[124] Arunakumara K.K.I.U., Walpola B.C., Yoon M.H. (2015). Bioaugmentation-assisted phytoextraction of Co, Pb and Zn: An assessment with a phosphate-solubilizing bacterium isolated from metal-contaminated mines of Boryeong area in South Korea. Biotechnol. Agron. Soc. Environ..

[125] Chakraborty S., Das S., Banerjee S., Mukherjee S., Ganguli A., Mondal S. (2021). Heavy metals bio-removal potential of the isolated *Klebsiella* Sp TIU20 strain which improves growth of economic crop plant (*Vigna radiata* L.) under heavy metals stress by exhibiting plant growth promoting and protecting traits. Biocatal. Agric. Biotechnol..

[126] Mallick I., Bhattacharyya C., Mukherji S., Dey D., Sarkar S.C., Mukhopadhyay U.K., Ghosh A. (2018). Effective rhizoinoculation and biofilm formation by arsenic immobilizing halophilic plant growth promoting bacteria (PGPB) Isolated from mangrove rhizosphere: A step towards arsenic rhizoremediation. Sci. Total Environ..

[127] Zanganeh F., Heidari A., Sepehr A., Rohani A. (2022). Bioaugmentation and bioaugmentation–assisted phytoremediation of heavy metal contaminated soil by a synergistic effect of cyanobacteria inoculation, biochar, and purslane (*Portulaca oleracea* L.). Environ. Sci. Pollut. Res..

[128] Atigh Z.B.Q., Heidari A., Sepehr A., Bahreini M., Mahbub K.R. (2020). Bioremediation of heavy metal contaminated soils originated from iron ore mine by bio-augmentation with native cyanobacteria. Iran. J. Energy Environ..

[129] Bilal S., Shahzad R., Imran M., Jan R., Kim K.M., Lee I.J. (2020). Synergistic association of endophytic fungi enhances *Glycine max* L. resilience to combined abiotic stresses: Heavy metals, high temperature and drought stress. Ind. Crops Prod..

[130] Paredes-Páliz K., Rodríguez-Vázquez R., Duarte B., Caviedes M.A., Mateos-Naranjo E., Redondo-Gómez S., Caçador M.I., Rodríguez-Llorente I.D., Pajuelo E. (2018). Investigating the mechanisms underlying phytoprotection by plant growth-promoting rhizobacteria in *Spartina densiflora* under metal stress. Plant Biol..

[131] Ashraf S., Afzal M., Naveed M., Shahid M., Zahir Z.A. (2018). Endophytic bacteria enhance remediation of tannery effluent in constructed wetlands vegetated with *Leptochloa fusca*. Int. J. Phytoremed..

[132] Xie Y., Bu H., Feng Q., Wassie M., Amee M., Jiang Y., Bi Y., Hu L., Chen L. (2021). Identification of Cd-resistant microorganisms from heavy metal-contaminated soil and its potential in promoting the growth and cd accumulation of bermudagrass. Environ. Res..

[133] Rahman S., Khalid M., Kayani S.I., Tang K. (2020). The ameliorative effects of exogenous inoculation of *Piriformospora indica* on molecular, biochemical and physiological parameters of *Artemisia annua* L. under arsenic stress condition. Ecotoxicol. Environ. Saf..

[134] Li D., Zheng X., Lin L., An Q., Jiao Y., Li Q., Li Z., Hong Y., Zhang K., Xie C. (2022). Remediation of soils co-contaminated with cadmium and dichlorodiphenyltrichloroethane by king grass associated with *Piriformospora indica*: Insights into the regulation of root excretion and reshaping of rhizosphere microbial community structure. J. Hazard. Mater..

[135] Khan N., Bano A. (2016). Modulation of Phytoremediation and Plant Growth by the Treatment with PGPR, Ag nanoparticle and untreated municipal wastewater. Int. J. Phytoremed..

[136] Rizvi A., Khan M.S. (2017). Biotoxic impact of heavy metals on growth, oxidative stress and morphological changes in root structure of wheat (*Triticum aestivum* L.) and stress alleviation by *Pseudomonas aeruginosa* strain CPSB1. Chemosphere.

[137] Kaur P., Singh S., Kumar V., Singh N., Singh J. (2018). Effect of rhizobacteria on arsenic uptake by macrophyte *Eichhornia crassipes* (Mart.) solms. Int. J. Phytoremed..

[138] Chen B., Luo S., Wu Y., Ye J., Wang Q., Xu X. (2017). The effects of the endophytic bacterium *Pseudomonas fluorescens* Sasm05 and IAA on the plant growth and cadmium uptake of *Sedum alfredii* Hance. Front. Microbiol..

[139] Shabaan M., Asghar H.N., Akhtar M.J., Ali Q., Ejaz M. (2021). Role of plant growth promoting rhizobacteria in the alleviation of lead toxicity to *Pisum sativum* L.. Int. J. Phytoremed..

[140] Ma Y., Rajkumar M., Oliveira R.S., Zhang C., Freitas H. (2019). Potential of Plant Beneficial Bacteria and Arbuscular Mycorrhizal Fungi in Phytoremediation of Metal-Contaminated Saline Soils. J. Hazard. Mater..

[141] Tirry N., Kouchou A., El Omari B., Ferioun M., El Ghachtouli N. (2021). Improved chromium tolerance of *Medicago sativa* by plant growth-promoting rhizobacteria (PGPR). J. Genet. Eng. Biotechnol..

[142] Montalbán B., Thijs S., Lobo M.C., Weyens N., Ameloot M., Vangronsveld J., Pérez-Sanz A. (2017). Cultivar and metal-specific effects of endophytic bacteria in *Helianthus tuberosus* exposed to Cd and Zn. Int. J. Mol. Sci..

[143] Karimi A., Khodaverdiloo H., Rasouli-Sadaghiani M.H. (2018). Microbial-enhanced phytoremediation of lead contaminated calcareous soil by *Centaurea cyanus* L.. CLEAN—Soil Air Water.

[144] Mishra V., Gupta A., Kaur P., Singh S., Singh N., Gehlot P., Singh J. (2016). Synergistic effects of arbuscular mycorrhizal fungi and plant growth promoting rhizobacteria in bioremediation of iron contaminated soils. Int. J. Phytoremed..

[145] Sharma S., Anand G., Singh N., Kapoor R. (2017). Arbuscular mycorrhiza augments arsenic tolerance in wheat (*Triticum aestivum* L.) by strengthening antioxidant defense system and thiol metabolism. Front. Plant Sci..

[146] Singh G., Pankaj U., Chand S., Verma R.K. (2019). Arbuscular mycorrhizal fungi-assisted phytoextraction of toxic metals by *Zea mays* L. from tannery sludge. Soil Sediment Contam..

[147] Zhang X.F., Hu Z.H., Yan T.X., Lu R.R., Peng C.L., Li S.S., Jing Y.X. (2019). Arbuscular mycorrhizal fungi alleviate cd phytotoxicity by altering Cd subcellular distribution and chemical forms in *Zea mays*. Ecotoxicol. Environ. Saf..

[148] Peng W., Li X., Song J., Jiang W., Liu Y., Fan W. (2018). Bioremediation of cadmium- and zinc-contaminated soil using *Rhodobacter sphaeroides*. Chemosphere.

[149] Carlos M.H.J., Stefani P.V.Y., Janette A.M., Melani M.S.S., Gabriela P.O. (2016). Assessing the effects of heavy metals in ACC deaminase and IAA production on plant growth-promoting bacteria. Microbiol. Res..

[150] Jin Z., Deng S., Wen Y., Jin Y., Pan L., Zhang Y., Black T., Jones K.C., Zhang H., Zhang D. (2019). Application of *Simplicillium chinense* for Cd and Pb biosorption and enhancing heavy metal phytoremediation of soils. Sci. Total Environ..

[151] Sepehri M., Khatabi B. (2021). Combination of siderophore-producing bacteria and *Piriformospora indica* provides an efficient approach to improve cadmium tolerance in alfalfa. Microb. Ecol..

[152] Seifikalhor M., Hassani S.B., Aliniaeifard S. (2020). Seed priming by cyanobacteria (*Spirulina platensis*) and salep gum enhances tolerance of maize plant against cadmium toxicity. J. Plant Growth Regul..

[153] Cao S., Wang W., Zhao Y., Yang S., Wang F., Zhang J., Sun Y. (2016). Enhancement of lead phytoremediation by perennial ryegrass (*Lolium perenne* L.) using agent of *Streptomyces pactum* Act12. J. Pet. Environ. Biotechnol..

[154] Jeyasundar P.G.S.A., Ali A., Azeem M., Li Y., Guo D., Sikdar A., Abdelrahman H., Kwon E., Antoniadis V., Mani V.M. (2021). Green remediation of toxic metals contaminated mining soil using bacterial consortium and *Brassica juncea*. Environ. Pollut..

[155] El-Shahir A.A., El-Tayeh N.A., Ali O.M., Abdel Latef A.A.H., Loutfy N. (2021). The Effect of endophytic *Talaromyces pinophilus* on growth, absorption and accumulation of heavy metals of *Triticum aestivum* grown on sandy soil amended by sewage sludge. Plants.

[156] Malik A., Butt T.A., Naqvi S.T.A., Yousaf S., Qureshi M.K., Zafar M.I., Farooq G., Nawaz I., Iqbal M. (2020). Lead tolerant endophyte *Trametes hirsuta* Improved the growth and lead accumulation in the vegetative parts of *Triticum aestivum* L.. Heliyon.

[157] Li X., Zhang X., Wang X., Yang X., Cui Z. (2019). Bioaugmentation-assisted phytoremediation of lead and salinity co-contaminated soil by *Suaeda salsa* and *Trichoderma asperellum*. Chemosphere.

[158] Belimov A.A., Shaposhnikov A.I., Azarova T.S., Makarova N.M., Safronova V.I., Litvinskiy V.A., Nosikov V.V., Zavalin A.A., Tikhonovich I.A. (2020). Microbial consortium of pgpr, rhizobia and arbuscular mycorrhizal fungus makes pea mutant SGECdt comparable with indian mustard in cadmium tolerance and accumulation. Plants.

[159] Berg G., Rybakova D., Grube M., Köberl M. (2016). The plant microbiome explored: Implications for experimental botany. J. Exp. Bot..

[160] Thijs S., Sillen W., Rineau F., Weyens N., Vangronsveld J. (2016). Towards an enhanced understanding of plant-microbiome interactions to improve phytoremediation: Engineering the metaorganism. Front. Microbiol..

[161] Vryzas Z. (2016). The plant as metaorganism and research on next-generation systemic pesticides—Prospects and challenges. Front. Microbiol..

[162] Ahkami A.H., Allen White R., Handakumbura P.P., Jansson C. (2017). Rhizosphere engineering: Enhancing sustainable plant ecosystem productivity. Rhizosphere.

[163] Ahmadi K., Zarebanadkouki M., Ahmed M.A., Ferrarini A., Kuzyakov Y., Kostka S.J., Carminati A. (2017). Rhizosphere engineering: Innovative Improvement of root environment. Rhizosphere.

[164] Bell T.H., Cloutier-Hurteau B., Al-Otaibi F., Turmel M.C., Yergeau E., Courchesne F., St-Arnaud M. (2015). Early rhizosphere microbiome composition is related to the growth and Zn uptake of willows introduced to a former landfill. Environ. Microbiol..

[165] Johnson N.C., Wilson G.W.T., Bowker M.A., Wilson J.A., Miller R.M. (2010). Resource limitation is a driver of local adaptation in mycorrhizal symbioses. Proc. Natl. Acad. Sci. USA.

[166] Philippot L., Raaijmakers J.M., Lemanceau P., Van Der Putten W.H. (2013). Going Back to the Roots: The microbial ecology of the rhizosphere. Nat. Rev. Microbiol..

[167] Sasse J., Martinoia E., Northen T. (2018). Feed your friends: Do plant exudates shape the root microbiome?. Trends Plant Sci..

[168] Tian T., Reverdy A., She Q., Sun B., Chai Y. (2020). The role of rhizodeposits in shaping rhizomicrobiome. Environ. Microbiol. Rep..

[169] Abdu N., Abdullahi A.A., Abdulkadir A. (2017). Heavy metals and soil microbes. Environ. Chem. Lett..

[170] Berg J., Brandt K.K., Al-Soud W.A., Holm P.E., Hansen L.H., Sørensen S.J., Nybroe O. (2012). Selection for Cu-tolerant bacterial communities with altered composition, but unaltered richness, via long-term cu exposure. Appl. Environ. Microbiol..

[171] Gołebiewski M., Deja-Sikora E., Cichosz M., Tretyn A., Wróbel B. (2014). 16S rDNA pyrosequencing analysis of bacterial community in heavy metals polluted soils. Microb. Ecol..

[172] Sobolev D., Begonia M.F.T. (2008). Effects of heavy metal contamination upon soil microbes: Lead-induced changes in general and denitrifying microbial communities as evidenced by molecular markers. Int. J. Environ. Res. Public Health.

[173] Yergeau E., Sanschagrin S., Maynard C., St-Arnaud M., Greer C.W. (2014). Microbial expression profiles in the rhizosphere of willows depend on soil contamination. ISME J..

[174] Chen J., Li J., Zhang H., Shi W., Liu Y. (2019). Bacterial heavy-metal and antibiotic resistance genes in a copper tailing dam area in northern China. Front. Microbiol..

[175] Bell T.H., Joly S., Pitre F.E., Yergeau E. (2014). Increasing phytoremediation efficiency and reliability using novel omics approaches. Trends Biotechnol..

[176] Schenk P.M., Carvalhais L.C., Kazan K. (2012). Unraveling plant-microbe interactions: Can multi-species transcriptomics help?. Trends Biotechnol..

[177] Jagtap U.B., Bapat V.A. (2015). Genetic engineering of plants for heavy metal removal from soil. Heavy Metal Contamination of Soils.

[178] Lee J.H. (2013). An overview of phytoremediation as a potentially promising technology for environmental pollution control. Biotechnol. Bioprocess Eng..

[179] Faucon M.P., Houben D., Lambers H. (2017). Plant Functional Traits: Soil and Ecosystem Services. Trends Plant Sci..

[180] Faucon M.P. (2020). Plant–Soil Interactions as Drivers of the Structure and Functions of Plant Communities. Diversity.

[181] Kapahi M., Sachdeva S. (2019). Bioremediation Options for Heavy Metal Pollution. J. Health Pollut..

[182] Sun W., Cheng K., Sun K.Y., Ma X. (2021). Microbially mediated remediation of contaminated sediments by heavy metals: A critical review. Curr. Pollut. Rep..

[183] Njoku K.L., Akinyede O.R., Obidi O.F. (2020). Microbial remediation of heavy metals contaminated media by *Bacillus megaterium* and *Rhizopus stolonifer*. Sci. Afr..

[184] Achal V., Pan X., Fu Q., Zhang D. (2012). Biomineralization based remediation of As(III) contaminated soil by *Sporosarcina ginsengisoli*. J. Hazard. Mater..

[185] Imam S.S.A., Rajpoot I.K., Gajjar B., Sachdeva A. (2016). Comparative study of heavy metal bioremediation in soil by *Bacillus subtilis* and *Saccharomyces cerevisiae*. Indian J. Sci. Technol..

[186] Kang C.H., Kwon Y.J., So J.S. (2016). Bioremediation of heavy metals by using bacterial mixtures. Ecol. Eng..

[187] Chibuike G.U., Obiora S.C. (2014). Heavy metal polluted soils: Effect on plants and bioremediation methods. Appl. Environ. Soil Sci..

[188] Gupta A., Joia J. (2016). Microbes as potential tool for remediation of heavy metals: A review. J. Microb. Biochem. Technol..

[189] Hussain N., Abbasi T., Abbasi S.A. (2016). Vermiremediation of an invasive and pernicious weed salvinia (*Salvinia molesta*). Ecol. Eng..

[190] Mawang C.I., Azman A.S., Fuad A.S.M., Ahamad M. (2021). Actinobacteria: An eco-friendly and promising technology for the bioaugmentation of contaminants. Biotechnol. Rep..

[191] Fauziah S.H., Agamuthu P., Hashim R., Izyani A.K., Emenike C.U. (2017). Assessing the bioaugmentation potentials of individual isolates from landfill on metal-polluted soil. Environ. Earth Sci..

[192] Singh J.S., Abhilash P.C., Singh H.B., Singh R.P., Singh D.P. (2011). Genetically engineered bacteria: An emerging tool for environmental remediation and future research perspectives. Gene.

[193] Pant G., Garlapati D., Agrawal U., Prasuna R.G., Mathimani T., Pugazhendhi A. (2021). Biological approaches practised using genetically engineered microbes for a sustainable environment: A review. J. Hazard. Mater..

[194] Liu L., Bilal M., Duan X., Iqbal H.M.N. (2019). Mitigation of environmental pollution by genetically engineered bacteria—Current challenges and future perspectives. Sci. Total Environ..

[195] Verma S., Kuila A. (2019). Bioremediation of heavy metals by microbial process. Environ. Technol. Innov..

[196] Azad M.A.K., Amin L., Sidik N.M. (2014). Genetically engineered organisms for bioremediation of pollutants in contaminated sites. Chin. Sci. Bull..

[197] Zhu N., Zhang B., Yu Q. (2020). Genetic engineering-facilitated coassembly of synthetic bacterial cells and magnetic nanoparticles for efficient heavy metal removal. ACS Appl. Mater. Interfaces.

[198] Liu J., Zhu N., Zhang Y., Ren T., Shao C., Shi R., Li X., Ju M., Ma T., Yu Q. (2021). Transcription profiling-guided remodeling of sulfur metabolism in synthetic bacteria for efficiently capturing heavy metals. J. Hazard. Mater..

[199] Wang D., Zheng Y., Xu L., Fan X., Wei N., Jin N., Huang S., Xiao Q., Wu Z. (2019). Engineered cells for selective detection and remediation of Hg^2+^ based on transcription factor MerR regulated cell surface displayed systems. Biochem. Eng. J..

[200] Igiri B.E., Okoduwa S.I.R., Idoko G.O., Akabuogu E.P., Adeyi A.O., Ejiogu I.K. (2018). Toxicity and bioremediation of heavy metals contaminated ecosystem from tannery wastewater: A review. J. Toxicol..

[201] Bazzi W., Abou Fayad A.G., Nasser A., Haraoui L.P., Dewachi O., Abou-Sitta G., Nguyen V.K., Abara A., Karah N., Landecker H. (2020). Heavy metal toxicity in armed conflicts potentiates amr in *A. baumannii* by selecting for antibiotic and heavy metal co-resistance mechanisms. Front. Microbiol..

[202] Mishra J., Singh R., Arora N.K. (2017). Alleviation of heavy metal stress in plants and remediation of soil by rhizosphere microorganisms. Front. Microbiol..

[203] Yue Z.B., Li Q., Li C.C., Chen T.H., Wang J. (2015). Component analysis and heavy metal adsorption ability of extracellular polymeric substances (EPS) from sulfate reducing bacteria. Bioresour. Technol..

[204] Machuca Á., Rai M., Varma A. (2011). Metal-chelating agents from ectomycorrhizal fungi and their biotechnological potential. Diversity and Biotechnology of Ectomycorrhizae.

[205] Verma S., Bhatt P., Verma A., Mudila H., Prasher P., Rene E.R. (2021). Microbial technologies for heavy metal remediation: Effect of process conditions and current practices. Clean Technol. Environ. Policy.

[206] González-Chávez M.C., Carrillo-González R., Wright S.F., Nichols K.A. (2004). The role of glomalin, a protein produced by arbuscular mycorrhizal fungi, in sequestering potentially toxic elements. Environ. Pollut..

[207] Mishra S., Lin Z., Pang S., Zhang Y., Bhatt P., Chen S. (2021). Biosurfactant Is a powerful tool for the bioremediation of heavy metals from contaminated soils. J. Hazard. Mater..

[208] Lal S., Ratna S., Said O.B., Kumar R. (2018). Biosurfactant and exopolysaccharide-assisted rhizobacterial technique for the remediation of heavy metal contaminated soil: An advancement in metal phytoremediation technology. Environ. Technol. Innov..

[209] Ravindran A., Sajayan A., Priyadharshini G.B., Selvin J., Kiran G.S. (2020). Revealing the efficacy of thermostable biosurfactant in heavy metal bioremediation and surface treatment in vegetables. Front. Microbiol..

[210] Ayangbenro A.S., Babalola O.O. (2020). Genomic analysis of *Bacillus cereus* NWUAB01 and its heavy metal removal from polluted soil. Sci. Rep..

[211] Ahuekwe E.F., Okoli B.E., Stanley H.O., Kinigoma B. Evaluation of hydrocarbon emulsification and heavy metal detoxification potentials of sophorolipid biosurfactants produced from waste substrates using yeast and mushroom. Proceedings of the SPE African Health, Safety, Security, Environment, and Social Responsibility Conference and Exhibition.

[212] Gomaa E.Z., El-Meihy R.M. (2019). Bacterial biosurfactant from *Citrobacter freundii* MG812314.1 as a bioremoval tool of heavy metals from wastewater. Bull. Natl. Res. Cent..

[213] Sun G.L., Reynolds E.E., Belcher A.M. (2020). Using yeast to sustainably remediate and extract heavy metals from waste waters. Nat. Sustain..

[214] Xu Y.N., Chen Y. (2020). Advances in heavy metal removal by sulfate-reducing bacteria. Water Sci. Technol..

[215] Choi J.H., Kim I.H., Kim Y.K., Oh B.K. (2015). Effective bioremediation of cadmium (II), nickel (II), and chromium (VI) in a marine environment by using *Desulfovibrio desulfuricans*. Biotechnol. Bioprocess Eng..

[216] Rana K., Rana N., Singh B. (2020). Applications of sulfur oxidizing bacteria. Physiological and Biotechnological Aspects of Extremophiles.

[217] Wu X., Huang P., Dong C., Deng X. (2021). Nickel bioaccumulation by a marine bacterium *Brevibacterium* sp. (X6) isolated from shenzhen bay, China. Mar. Pollut. Bull..

[218] Oves M., Khan M.S., Qari H.A. (2017). *Ensifer adhaerens* for heavy metal bioaccumulation, biosorption, and phosphate solubilization under metal stress condition. J. Taiwan Inst. Chem. Eng..

[219] Hansda A., Kumar V., Anshumali (2016). A comparative review towards potential of microbial cells for heavy metal removal with emphasis on biosorption and bioaccumulation. World J. Microbiol. Biotechnol..

[220] Danouche M., El Ghachtouli N., El Arroussi H. (2021). Phycoremediation mechanisms of heavy metals using living green microalgae: Physicochemical and molecular approaches for enhancing selectivity and removal capacity. Heliyon.

[221] Shahpiri A., Mohammadzadeh A. (2018). Mercury Removal by Engineered *Escherichia coli* cells expressing different rice metallothionein isoforms. Ann. Microbiol..

[222] Yu Y., Shi K., Li X., Luo X., Wang M., Li L., Wang G., Li M. (2022). Reducing cadmium in rice using metallothionein surface-engineered bacteria WH16-1-MT. Environ. Res..

[223] Rehman A., Anjum M.S. (2011). Multiple metal tolerance and biosorption of cadmium by *Candida tropicalis* isolated from industrial effluents: Glutathione as detoxifying agent. Environ. Monit. Assess..

[224] Khullar S., Reddy M.S. (2020). Arsenic toxicity and its mitigation in ectomycorrhizal fungus *Hebeloma cylindrosporum* through glutathione biosynthesis. Chemosphere.

[225] Yong X., Chen Y., Liu W., Xu L., Zhou J., Wang S., Chen P., Ouyang P., Zheng T. (2014). Enhanced cadmium resistance and accumulation in *Pseudomonas putida* KT2440 expressing the phytochelatin synthase gene of *Schizosaccharomyces pombe*. Lett. Appl. Microbiol..

[226] Ahemad M. (2019). Remediation of metalliferous soils through the heavy metal resistant plant growth promoting bacteria: Paradigms and prospects. Arab. J. Chem..

[227] Sher S., Rehman A. (2019). Use of heavy metals resistant bacteria—A strategy for arsenic bioremediation. Appl. Microbiol. Biotechnol..

[228] Banerjee S., Datta S., Chattyopadhyay D., Sarkar P. (2011). Arsenic accumulating and transforming bacteria isolated from contaminated soil for potential use in bioremediation. J. Environ. Sci. Health—Part A Toxic/Hazard. Subst. Environ. Eng..

[229] Sharma S., Adholeya A. (2011). Detoxification and accumulation of chromium from tannery effluent and spent chrome effluent by *Paecilomyces lilacinus* Fungi. Int. Biodeterior. Biodegrad..

[230] Narayani M., Shetty K.V. (2013). Chromium-resistant bacteria and their environmental condition for hexavalent chromium removal: A review. Crit. Rev. Environ. Sci. Technol..

[231] Batool R., Yrjälä K., Hasnain S. (2012). Hexavalent chromium reduction by bacteria from tannery effluent. J. Microbiol. Biotechnol..

[232] Wiatrowski H.A., Ward P.M., Barkay T. (2006). Novel reduction of mercury(II) by mercury-sensitive dissimilatory metal reducing bacteria. Environ. Sci. Technol..

[233] Bolan N., Kunhikrishnan A., Thangarajan R., Kumpiene J., Park J., Makino T., Beth M., Scheckel K. (2014). Remediation of heavy metal (loid)s contaminated soils—To mobilize or to immobilize?. J. Hazard. Mater..

[234] Di X., Beesley L., Zhang Z., Zhi S., Jia Y., Ding Y. (2019). Microbial arsenic methylation in soil and uptake and metabolism of methylated arsenic in plants: A review. Int. J. Environ. Res. Public Health.

[235] Verma S., Verma P.K., Chakrabarty D. (2019). Arsenic bio-volatilization by engineered yeast promotes rice growth and reduces arsenic accumulation in grains. Int. J. Environ. Res..

[236] Hu H., Lin H., Zheng W., Tomanicek S.J., Johs A., Feng X., Elias D.A., Liang L., Gu B. (2013). Oxidation and methylation of dissolved elemental mercury by anaerobic bacteria. Nat. Geosci..

[237] Soares Guimarães L.H., Segura F.R., Tonani L., Von-Zeska-Kress M.R., Rodrigues J.L., Calixto L.A., Silva F.F., Batista B.L. (2019). Arsenic volatilization by *Aspergillus* sp. and *Penicillium* sp. isolated from rice rhizosphere as a promising eco-safe tool for arsenic mitigation. J. Environ. Manag..

[238] Bhattacharya A., Gupta A., Kaur A., Malik D. (2014). Efficacy of *Acinetobacter* sp. B9 for simultaneous removal of phenol and hexavalent chromium from co-contaminated system. Appl. Microbiol. Biotechnol..

[239] De J., Ramaiah N., Vardanyan L. (2008). Detoxification of toxic heavy metals by marine bacteria highly resistant to mercury. Mar. Biotechnol..

[240] Sharma B., Shukla P. (2021). Lead bioaccumulation mediated by *Bacillus cereus* BPS-9 from an industrial waste contaminated site encoding heavy metal resistant genes and their transporters. J. Hazard. Mater..

[241] Chaturvedi M.K. (2011). Studies on chromate removal by chromium-resistant *Bacillus* sp. isolated from tannery effluent. J. Environ. Prot..

[242] Salehizadeh H., Shojaosadati S.A. (2003). Removal of metal ions from aqueous solution by polysaccharide produced from *Bacillus firmus*. Water Res..

[243] Muneer B., Iqbal M.J., Shakoori F.R., Shakoori A.R. (2013). Tolerance and biosorption of mercury by microbial consortia: Potential use in bioremediation of wastewater. Pak. J. Zool..

[244] Fathima Benazir J., Suganthi R., Rajvel D., Padmini Pooja M., Mathithumilan B. (2010). Bioremediation of Chromium in Tannery Effluent by Microbial Consortia. Afr. J. Biotechnol..

[245] Mohapatra R.K., Parhi P.K., Pandey S., Bindhani B.K., Thatoi H., Panda C.R. (2019). Active and passive biosorption of Pb(II) using live and dead biomass of marine bacterium *Bacillus xiamenensis* PbRPSD202: Kinetics and isotherm studies. J. Environ. Manag..

[246] Bharagava R.N., Mishra S. (2018). Hexavalent chromium reduction potential of *Cellulosimicrobium* sp. isolated from common effluent treatment plant of tannery industries. Ecotoxicol. Environ. Saf..

[247] Kim I.H., Choi J.H., Joo J.O., Kim Y.K., Choi J.W., Oh B.K. (2015). Development of a microbe-zeolite carrier for the effective elimination of heavy metals from seawater. J. Microbiol. Biotechnol..

[248] Congeevaram S., Dhanarani S., Park J., Dexilin M., Thamaraiselvi K. (2007). Biosorption of chromium and nickel by heavy metal resistant fungal and bacterial isolates. J. Hazard. Mater..

[249] Juwarkar A.A., Nair A., Dubey K.V., Singh S.K., Devotta S. (2007). Biosurfactant technology for remediation of cadmium and lead contaminated soils. Chemosphere.

[250] Zhao R., Wang B., Cai Q.T., Li X.X., Liu M., Hu D., Guo D.B., Wang J., Fan C. (2016). Bioremediation of hexavalent chromium pollution by *Sporosarcina saromensis* M52 isolated from offshore sediments in Xiamen, China. Biomed. Environ. Sci..

[251] Sedlakova-Kadukova J., Kopcakova A., Gresakova L., Godany A., Pristas P. (2019). Bioaccumulation and biosorption of zinc by a novel *Streptomyces* K11 strain isolated from highly alkaline aluminium brown mud disposal site. Ecotoxicol. Environ. Saf..

[252] Luna J.M., Rufino R.D., Sarubbo L.A. (2016). Biosurfactant from *Candida sphaerica* UCP0995 exhibiting heavy metal remediation properties. Process Saf. Environ. Prot..

[253] Basak G., Das N. (2014). Characterization of sophorolipid biosurfactant produced by *Cryptococcus* sp. VITGBN2 and its application on Zn(II) removal from electroplating wastewater. J. Environ. Biol..

[254] Chatterjee S., Chatterjee N.C., Dutta S. (2012). Bioreduction of chromium (VI) to chromium (III) by a novel yeast strain *Rhodotorula mucilaginosa* (MTCC 9315). Afr. J. Biotechnol..

[255] Bano A., Hussain J., Akbar A., Mehmood K., Anwar M., Hasni M.S., Ullah S., Sajid S., Ali I. (2018). Biosorption of heavy metals by obligate halophilic fungi. Chemosphere.

[256] Amini M., Younesi H., Bahramifar N. (2009). Biosorption of nickel(II) from aqueous solution by *Aspergillus niger*: Response surface methodology and isotherm study. Chemosphere.

[257] Taştan B.E., Ertuǧrul S., Dönmez G. (2010). Effective bioremoval of reactive dye and heavy metals by *Aspergillus versicolor*. Bioresour. Technol..

[258] Noormohamadi H.R., Fat’hi M.R., Ghaedi M., Ghezelbash G.R. (2019). Potentiality of white-rot fungi in biosorption of nickel and cadmium: Modeling optimization and kinetics study. Chemosphere.

[259] Abd El-Hameed M.M., Abuarab M.E., Abdel Mottaleb S., El-Bahbohy R.M., Bakeer G.A. (2018). Comparative studies on growth and Pb(II) removal from aqueous solution by *Nostoc muscorum* and *Anabaena variabilis*. Ecotoxicol. Environ. Saf..

[260] Shen L., Saky S.A., Yang Z., Ho S.H., Chen C., Qin L., Zhang G., Wang Y., Lu Y. (2019). The critical utilization of active heterotrophic microalgae for bioremoval of Cr(VI) in organics co-contaminated wastewater. Chemosphere.

[261] Chandrashekharaiah P., Debanjan S., Santanu D., Avishek B. (2021). Cadmium biosorption and biomass production by two freshwater microalgae *Scenedesmus acutus* and *Chlorella pyrenoidosa*: An integrated approach. Chemosphere.

[262] Pradhan D., Sukla L.B., Mishra B.B., Devi N. (2019). Biosorption for removal of hexavalent chromium using microalgae *Scenedesmus* sp.. J. Clean. Prod..

[263] Mane P.C., Bhosle A.B. (2012). Bioremoval of some metals by living algae *Spirogyra* sp. and *Spirullina* sp. from aqueous solution. Int. J. Environ. Res..

[264] Cepoi L., Zinicovscaia I., Rudi L., Chiriac T., Miscu V., Djur S., Strelkova L., Grozdov D. (2020). *Spirulina platensis* as renewable accumulator for heavy metals accumulation from multi-element synthetic effluents. Environ. Sci. Pollut. Res..

[265] Xie Y., Fan J., Zhu W., Amombo E., Lou Y., Chen L., Fu J. (2016). Effect of heavy metals pollution on soil microbial diversity and bermudagrass genetic variation. Front. Plant Sci..

[266] Fajardo C., Costa G., Nande M., Botías P., García-Cantalejo J., Martín M. (2019). Pb, Cd, and Zn soil contamination: Monitoring functional and structural impacts on the microbiome. Appl. Soil Ecol..

[267] Li X., Meng D., Li J., Yin H., Liu H., Liu X., Cheng C., Xiao Y., Liu Z., Yan M. (2017). Response of soil microbial communities and microbial interactions to long-term heavy metal contamination. Environ. Pollut..

[268] Qiao L., Liu X., Zhang S., Zhang L., Li X., Hu X., Zhao Q., Wang Q., Yu C. (2021). Distribution of the microbial community and antibiotic resistance genes in farmland surrounding gold tailings: A metagenomics approach. Sci. Total Environ..

[269] Song J., Shen Q., Wang L., Qiu G., Shi J., Xu J., Brookes P.C., Liu X. (2018). Effects of Cd, Cu, Zn and their combined action on microbial biomass and bacterial community structure. Environ. Pollut..

[270] Zhang S., Yang G., Hou S., Zhang T., Li Z., Liang F. (2018). Distribution of ARGs and MGEs among glacial soil, permafrost, and sediment using metagenomic analysis. Environ. Pollut..

[271] Feng G., Xie T., Wang X., Bai J., Tang L., Zhao H., Wei W., Wang M., Zhao Y. (2018). Metagenomic Analysis of Microbial Community and Function Involved in Cd-Contaminated Soil. BMC Microbiol..

[272] Jiang X., Liu W., Xu H., Cui X., Li J., Chen J., Zheng B. (2021). Characterizations of heavy metal contamination, microbial community, and resistance genes in a tailing of the largest copper mine in China. Environ. Pollut..

[273] Hemmat-Jou M.H., Safari-Sinegani A.A., Mirzaie-Asl A., Tahmourespour A. (2018). Analysis of microbial communities in heavy metals-contaminated soils using the metagenomic approach. Ecotoxicology.

[274] Misson B., Garnier C., Lauga B., Dang D.H., Ghiglione J.F., Mullot J.U., Duran R., Pringault O. (2016). Chemical multi-contamination drives benthic prokaryotic diversity in the anthropized Toulon Bay. Sci. Total Environ..

[275] Li J., Hu H.W., Ma Y.B., Wang J.T., Liu Y.R., He J.Z. (2015). Long-term nickel exposure altered the bacterial community composition but not diversity in two contrasting agricultural soils. Environ. Sci. Pollut. Res..

[276] Huang C.C., Liang C.M., Yang T.I., Chen J.L., Wang W.K. (2021). Shift of bacterial communities in heavy metal-contaminated agricultural land during a remediation process. PLoS ONE.

[277] Wu Z., Yu F., Sun X., Wu S., Li X., Liu T., Li Y. (2018). Long term effects of *Lespedeza bicolor* revegetation on soil bacterial communities in Dexing copper mine tailings in Jiangxi Province, China. Appl. Soil Ecol..

[278] Li Y., Chen L., Wen H. (2015). Changes in the composition and diversity of bacterial communities 13 years after soil reclamation of abandoned mine land in eastern china. Ecol. Res..

[279] Moynahan O.S., Zabinski C.A., Gannon J.E. (2002). Microbial community structure and carbon-utilization diversity in a mine tailings revegetation study. Restor. Ecol..

[280] Zhang L., Liu W., Liu S., Zhang P., Ye C., Liang H. (2020). Revegetation of a barren rare earth mine using native plant species in reciprocal plantation: Effect of phytoremediation on soil microbiological communities. Environ. Sci. Pollut. Res..

[281] Chen Y.X., Wang Y.P., Wu W.X., Lin Q., Xue S.G. (2006). Impacts of chelate-assisted phytoremediation on microbial community composition in the rhizosphere of a copper accumulator and non-accumulator. Sci. Total Environ..

[282] Liu S., Liu W., Yang M., Zhou L., Liang H. (2016). The genetic diversity of soil bacteria affected by phytoremediation in a typical barren rare earth mined site of south china. Springer Plus.

[283] Wei Z., Hao Z., Li X., Guan Z., Cai Y., Liao X. (2019). The effects of phytoremediation on soil bacterial communities in an abandoned mine site of rare earth elements. Sci. Total Environ..

[284] Gupta G.S., Yadav G., Tiwari S., Upadhyay A.K., Singh R., Singh D.P. (2020). Restoration of Wetland Ecosystem: A Trajectory Towards a Sustainable Environment.

[285] Sayqal A., Ahmed O.B. (2021). Advances in Heavy Metal Bioremediation: An Overview. Appl. Bionics Biomech..

[286] Abatenh E., Gizaw B., Tsegaye Z., Wassie M. (2017). Application of microorganisms in bioremediation-review. J. Environ. Microbiol..

[287] Liu S., Zhang F., Chen J., Sun G. (2011). Arsenic removal from contaminated soil via biovolatilization by genetically engineered bacteria under laboratory conditions. J. Environ. Sci..

[288] Bruneel O., Mghazli N., Sbabou L., Héry M., Casiot C., Filali-Maltouf A. (2019). Role of microorganisms in rehabilitation of mining sites, focus on sub-Saharan African countries. J. Geochem. Explor..

